# Preservation of naive-phenotype CD4^+^ T cells after vaccination contributes to durable immunity

**DOI:** 10.1172/jci.insight.180667

**Published:** 2024-06-11

**Authors:** Yi-Gen Pan, Laurent Bartolo, Ruozhang Xu, Bijal V. Patel, Veronika I. Zarnitsyna, Laura F. Su

**Affiliations:** 1 Department of Medicine, Division of Rheumatology, Perelman School of Medicine, University of Pennsylvania, Philadelphia, Pennsylvania, USA.; 2 Corporal Michael J. Crescenz VA Medical Center, Philadelphia, Pennsylvania, USA.; 3 Department of Microbiology and Immunology, Emory University, Atlanta, Georgia, USA.

**Keywords:** Immunology, Vaccines, Memory, T cells

## Abstract

Memory T cells are conventionally associated with durable recall responses. In our longitudinal analyses of CD4^+^ T cell responses to the yellow fever virus (YFV) vaccine by peptide-MHC tetramers, we unexpectedly found CD45RO^–^CCR7^+^ virus-specific CD4^+^ T cells that expanded shortly after vaccination and persisted months to years after immunization. Further phenotypic analyses revealed the presence of stem cell–like memory T cells within this subset. In addition, after vaccination T cells lacking known memory markers and functionally resembling genuine naive T cells were identified, referred to herein as marker-negative T (T_MN_) cells. Single-cell TCR sequencing detected expanded clonotypes within the T_MN_ subset and identified T_MN_ TCRs shared with memory and effector T cells. Longitudinal tracking of YFV-specific responses over subsequent years revealed superior stability of T_MN_ cells, which correlated with the longevity of the overall tetramer^+^ population. These findings uncover additional complexity within the post-immune T cell compartment and implicate T_MN_ cells in durable immune responses.

## Abstract 2

Naive CD4^+ ^T cells persist after vaccination and may contribute to durable protective immunity.

## Introduction

Functional immunological memory underlies the protective efficacy of vaccines against subsequent infections (1, 2). However, the reason protection from some vaccines lasts decades whereas protection from others wanes after a few months remains unknown. A crucial aspect of immune memory involves CD4^+^ T cells (3). CD4^+^ T cells provide key signals for B cell maturation and high-affinity antibody production (4). They are also needed to support the expansion and maintenance of functional CD8^+^ T cells and can directly contribute to antiviral effects (4–6). Past studies in mice and humans have identified naive-like antigen-experienced T cells with superior longevity and plasticity as a source of durable memory (7–9). Broadly categorized as stem cell–like memory T (T_SCM_) cells, these cells phenotypically resemble naive T cells by positive CCR7 and CD45RA or negative CD45RO expression, yet they display differentiation markers such as CD95, CXCR3, and CD49d (9–11). In people immunized with the highly efficacious and durable yellow fever virus (YFV) vaccine, class I tetramer analyses identified T_SCM_ as the predominant phenotype of virus-specific CD8^+^ T cells greater than 8 years after vaccination (10, 12).

The durability of CD4^+^ T cell memory is less understood. Although capable of differentiating into T_SCM_ cells (13–16), CD4^+^ T cells are generally less responsive to homeostatic cytokines IL-7 and IL-15 (17–19), which augment T_SCM_ differentiation in cultured CD8^+^ T cells (20). Here, we examined virus-specific CD4^+^ T cells after YFV vaccination to delineate key features of durable CD4^+^ T cell responses. YFV-specific CD4^+^ T cells were identified and tracked longitudinally by direct ex vivo class II peptide-MHC (pMHC) tetramer staining. We showed the presence of various memory subsets within YFV-specific CD4^+^ T cells several months after vaccination, including T_SCM_ cells. Unexpectedly, about a quarter of CD45RO^–^CCR7^+^ tetramer-labeled T cells lacked CD95, CXCR3, CD11a, and CD49d expression, distinguishing them from T_SCM_ cells. These marker-negative T (T_MN_) cells were a part of expanded clonotypes and shared TCR sequences with memory and effector T cells, suggesting in vivo antigen responses. T_MN_ cells showed minimal decay over years and correlated with the stability of tetramer^+^ populations. Our findings expand the current definition of antigen-experienced T cells to include those that retain an undifferentiated phenotype. These T_MN_ cells may contribute to durable immunity.

## Results

### Detection of naive-like CD4^+^ T cells after YFV vaccination.

**Figure 1:**
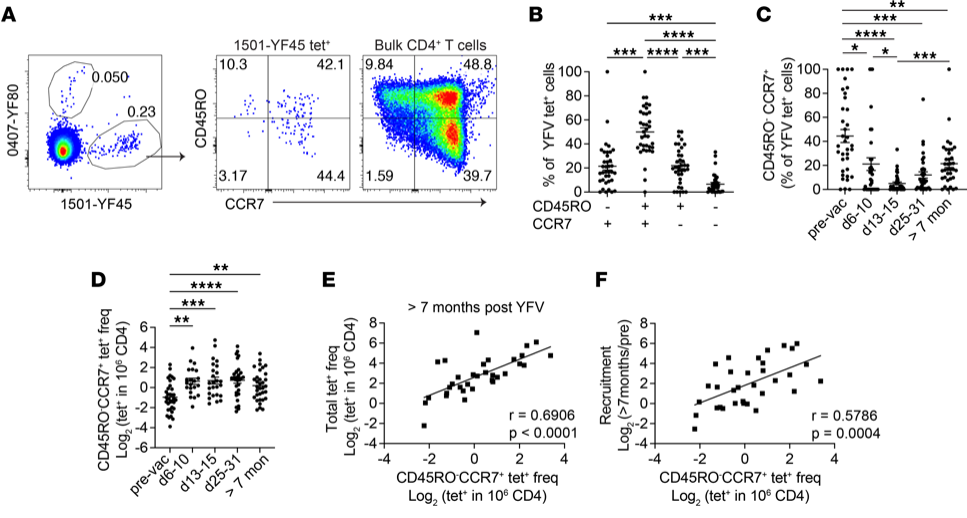
Identification of CD45RO^–^CCR7^+^ virus-specific CD4^+^ T cells after YFV vaccination. (**A**) Direct ex vivo tetramer and antibody staining of a representative YFV tetramer^+^ (tet^+^) population using blood collected about 7 months after YFV vaccination. (**B**) The percentage of YFV tet^+^ T cells with the indicated combination of CD45RO and CCR7 expression. Plot summarizes data from 36 specificities 7 to 34 months after YFV vaccination from 7 donors. (**C** and **D**) The abundance of CD45RO^–^CCR7^+^YFV tet^+^ CD4^+^ T cells in 7 healthy participants was quantified as a percentage of tetramer^+^ cells (**C**) or by frequency (**D**). Each symbol represents data from a distinct YFV-specific population. Experiments were repeated an average of 3.3 times. (**E**) The correlation between CD45RO^–^CCR7^+^YFV tet^+^ T cell frequency and the overall tet^+^ frequency of the same population at least 7 months after vaccination. (**F**) The correlation between the CD45RO^–^CCR7^+^YFV tet^+^ T cell frequency and the fold-change from the pre-vaccine baseline to the memory time point. CD45RO^–^CCR7^+^tet^+^ frequencies were determined using samples taken 7 to 34 months after vaccination. *n* = 33, and populations with no post-vaccine naive cells were excluded. Repeated measures (RM) 1-way ANOVA (**B**) or mixed effects analysis (**C** and **D**) was performed and corrected with Tukey’s multiple comparisons test. (**E** and **F**) Pearson’s correlation was computed. *P < 0.05, **P < 0.01, ***P < 0.001, ****P < 0.0001.

We had previously performed a longitudinal study of YFV-specific CD4^+^ T cells to evaluate the impact of the preexisting repertoire on T cell responses to primary immunization with the YFV vaccine (21). Starting with this data set, we examined features of memory T cells that developed at least 7 months after vaccination. This showed that approximately half of the YFV-specific memory pool consisted of central memory T (T_CM_) cells, with about 21% of tetramer^+^ cells retaining a naive-like CD45RO^–^CCR7^+^ phenotype (Figure 1, A and B). Proportionally, the abundance of CD45RO^–^CCR7^+^ subset was highest before vaccination, decreased initially after vaccination, and then reaccumulated several months later (Figure 1C). Quantified as cells per million CD4^+^ T cells, CD45RO^–^CCR7^+^ tetramer^+^ cells quickly increased and reached a peak approximately 1 month after vaccination (Figure 1D). The frequency of the CD45RO^–^CCR7^+^ T cell subset did not differ significantly by donor age and was associated with the robustness of the response (Supplemental Figure 1, A–C; supplemental material available online with this article; https://doi.org/10.1172/jci.insight.180667DS1). At the memory time point, CD45RO^–^CCR7^+^ YFV-specific T cells were more abundant in tetramer^+^ populations that reached a higher frequency and correlated with efficient recruitment of memory cells (Figure 1, E and F). These data suggest that the presence of CD45RO^–^CCR7^+^ CD4^+^ T cells is a feature of an effective T cell response.

### Post-immune T cells are heterogeneous and include a differentiation marker–negative subset.

**Figure 2:**
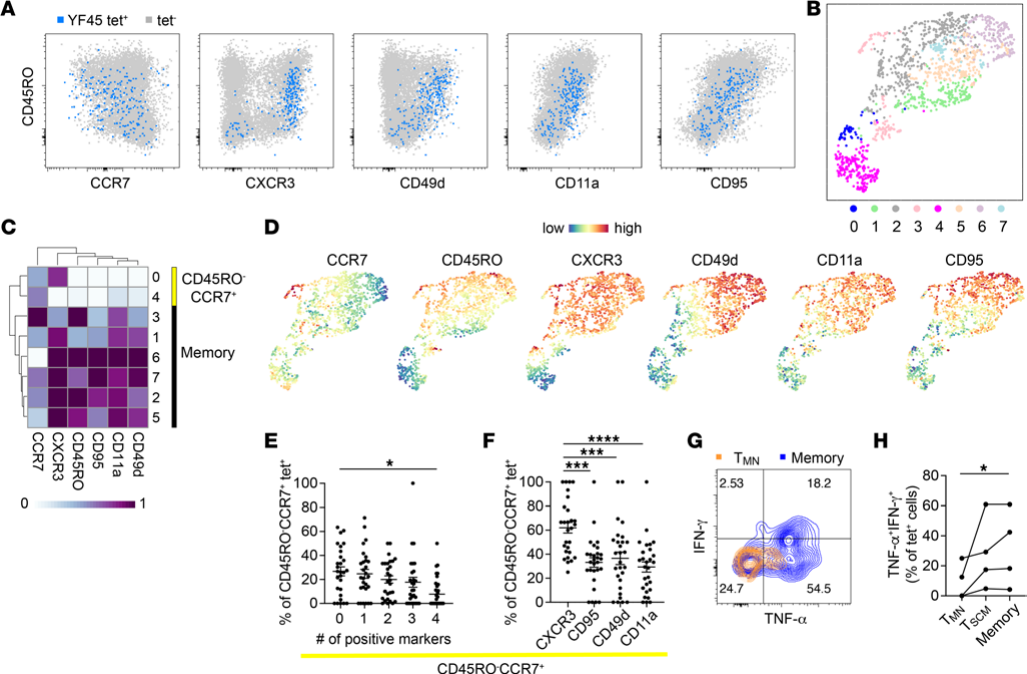
Post-vaccine CD4^+^ T cells are heterogeneous and include naive-like subsets. (**A**) FACS plots show the expression of the indicated marker on a representative YFV-specific population. The tet^+^ population is overlaid onto tet^–^ bulk CD4^+^ T cells. (**B**) UMAP displays phenograph-defined clusters. Data combine 1,465 CD4^+^ cells labeled by 7 YFV tetramers from HD3. (**C** and **D**) The staining intensity of individual markers is shown on a heatmap for each cluster (**C**) or displayed on the UMAP (**D**). (**E** and **F**) The relative abundance of CD45RO^–^CCR7^+^ YFV-specific T cells by the indicated numbers of markers (**E**) or the type of markers (**F**). Frequency in **F** combines all cells positive for a particular marker within the CD45RO^–^CCR7^+^ subset. Marker combinations were determined using Boolean operators on manually defined gates. Each symbol represents a tetramer^+^ population (*n* = 28). Experiments were repeated an average of 2.5 times. (**G**) PBMCs were stimulated for 4–5 hours by PMA and ionomycin and assayed for cytokine production by intracellular cytokine staining. The plot shows representative TNF-α and IFN-γ expression by T_MN_ cells and memory T cells (non-CD45RO^–^CD28^+^) from the same tetramer-labeled population. (**H**) T cell responses by TNF-α and IFN-γ production for the indicated phenotypic subset. Each population was identified with a pool of 5–7 tetramers of the same DR allele, using cells from 3 donors. (**E** and **F**) RM 1-way ANOVA was performed and corrected with Tukey’s multiple comparison test. (**H**) The Friedman test was performed and corrected using Dunn’s multiple comparison test. *P < 0.05, ***P < 0.001, ****P < 0.0001.

We hypothesized that the post-vaccine CD45RO^–^CCR7^+^ subset largely consisted of T_SCM_ cells as in CD8^+^ T cells (10, 12). To test this, we performed tetramer staining on 28 YFV-specific CD4^+^ populations from 7 individuals, recognizing 16 unique epitopes with antibodies against T_SCM_-associated markers, CXCR3, CD95, CD11a, and CD49d (Supplemental Tables 1 and 2). Staining with this broader antibody panel on blood collected 7 to 48 months after vaccination indeed identified CD45RO^–^CCR7^+^tetramer^+^ T cells that expressed 1 or more T_SCM_ markers. However, we noted that a portion of CD45RO^–^CCR7^+^ CD4^+^ T cells remained negative for CXCR3, CD95, CD11a, and CD49d expression (Figure 2A and Supplemental Figure 2, A–D). To gain further insights into the heterogeneity within the CD45RO^–^CCR7^+^ subset, we combined 1,465 YFV-specific CD4^+^ T cells from 1 donor and visualized combinatorial antibody staining on uniform manifold approximation and projection (UMAP) using the Spectre pipeline (22). This identified regions with low CD45RO and high CCR7 signals, which encompassed a CXCR3^+^ (cluster 0) and a T_SCM_ marker–negative population (cluster 4) (Figure 2, B–D). We defined CD45RO^–^CCR7^+^ cells lacking any measured differentiation markers as T_MN_ cells and classified those expressing at least 1 of CXCR3, CD95, CD11a, or CD49d as T_SCM_ cells (Supplemental Table 3). We then performed manual gating and used Boolean combinations of these gated cells to subdivide the CD45RO^–^CCR7^+^ subset into T_MN_ and various T_SCM_ combinations (Supplemental Figure 2, B–D). On average, 27% of the CD45RO^–^CCR7^+^ subset consisted of T_MN_ cells (Figure 2E). Among T_SCM_ cells, approximately a quarter expressed only 1 differentiation marker, with CXCR3 being the most common (Figure 2, E and F). Finding antigen-specific T cells that do not express known memory or T_SCM_ markers after a clear prior exposure was unexpected. To test if T_MN_ cells functionally behave like antigen-experienced T cells despite lacking surface markers of differentiation, we treated post-vaccine PBMCs with phorbol myristate acetate (PMA) and ionomycin for 4 to 5 hours. Antigen-specific T cells were captured by tetramers, divided into distinct phenotypic subsets, and analyzed for TNF-α and IFN-γ production. This showed that the post-immune T_MN_ subset produced significantly fewer cytokines compared with memory T cells within the same tetramer^+^ population (Figure 2, G and H). Thus, YFV vaccination induced a diverse post-immune repertoire that included CD4^+^ T_SCM_ cells and a naive-like T_MN_ population that lacked phenotypic and functional features of antigen experience.

### Virus-specific T_MN_ cells respond to antigens.

**Figure 3:**
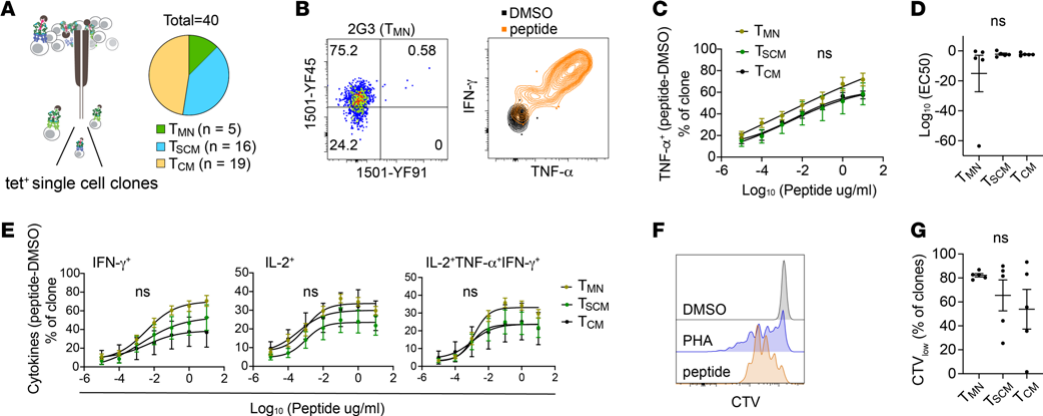
T_MN_-derived T cell clones respond to antigen stimulation. (**A**) Schematics of single-cell T cell cloning. Post-vaccine T cells from HD2 and HD3 were stained with 1501-YF45 tetramers; sorted based on T_MN_, T_SCM_, or T_CM_ phenotypes; and expanded for 2 to 3 weeks in culture. (**B**) In vitro–expanded T cell clones were restained with tetramers and cultured with vehicle- (DMSO) or peptide-treated monocyte-derived DCs. Representative plots show tetramer staining and cytokine production by intracellular cytokine staining. (**C** and **D**) T cell clones were stimulated with decreasing concentrations of YFV peptides. The response was measured by TNF-α production (**C**) and quantified by EC_50_ values after subtracting the background signal from vehicle-treated control (**D**). (**E**) Peptide dose response of T cell clones by IFN-γ, IL-2, and IL-2^+^TNF-α^+^IFN-γ^+^ production. (**F**) Representative histograms show CTV dilution in response to 10 μg/mL of peptide stimulation. (**G**) Plot summarizes the frequency of the CTV^lo^ population after a 5-day culture for clones in each phenotypic group. All experiments were repeated at least twice with *n* = 5 in each group. (**C** and **E**) RM 2-way ANOVA was performed and corrected with Tukey’s multiple comparison test. (**D** and **G**) Kruskal-Wallis test and Dunn’s multiple comparison test were used. PHA, phytohemagglutinin.

We were intrigued by the existence of virus-specific T cells that retained a naive functional phenotype after vaccination. Past studies have identified nonstimulatory TCR interactions that decoupled T cell activation from ligand binding (23). The impaired ability to respond productively to antigens may be one reason why some tetramer-labeled T cells retained a naive phenotype. To investigate this possibility, we quantified T_MN_, T_SCM_, and T_CM_ cells for differences in their functional avidity by peptide stimulation. YFV-specific T cell clones were generated using samples from 2 donors obtained 7 to 8 months after YFV vaccination. Among the 48 clones that grew, 40 clones (90%) had the correct specificity by tetramer restaining and/or response to peptides (Figure 3, A and B, and Supplemental Figure 3A). We did not identify peptide-nonresponsive T cells as all clones that were stained with tetramers responded to peptide stimulation. To determine if T_MN_ cells might be harder to activate because of a lower functional avidity, we divided the clones according to their direct ex vivo phenotype and selected 5 clones each from T_CM_, T_SCM_, and T_MN_ groups for further analyses. YFV-specific clones were stimulated with decreasing concentrations of the cognate peptide and analyzed for response by cytokine production (Supplemental Figure 3B). T_MN_ cell–derived clones responded similarly to peptides by TNF-α production, with no significant differences in maximal effective peptide concentration (EC_50_) values between groups (Figure 3, C and D). T cell clones, regardless of their ex vivo phenotypes, also produced similar levels of IFN-γ and IL-2 and had comparable TNF-α^+^IFN-γ^+^IL-2^+^ coexpression (Figure 3E). In addition, we evaluated the proliferative capacity of T_MN_, T_SCM_, and T_CM_ cell–derived clones by CellTrace Violet (CTV) dilution and observed no significant differences in the proliferative response to peptide stimulation (Figure 3, F and G, and Supplemental Figure 3C). Thus, TCR-ligand engagement is likely intact for vaccine-specific T cells that retained a naive phenotype after vaccination.

### Antigen-experienced T_MN_ cells.

**Figure 4:**
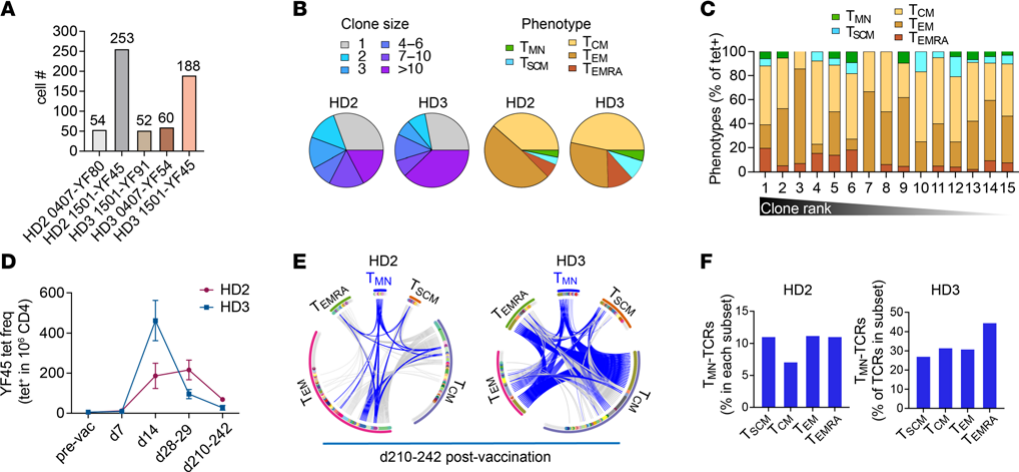
T_MN_ cells are clonally related to memory T cells. (**A**) The plot summarizes the number of cells sequenced from the indicated specificities and donors collected 242 (HD2) or 210 (HD3) days after primary YFV vaccination. (**B**) Clone size and phenotypic distribution of YFV tet^+^ populations in **A**. Phenotypic data were obtained by index sorting: T_MN_ (CD45RO^–^CCR7^+^CXCR3^–^CD95^–^CD11a^–^CD49d^–^), T_SCM_ (CD45RO^–^CCR7^+^ and positive for at least 1 of CXCR3, CD95, CD11a, or CD49d), T_CM_ (CD45RO^+^CCR7^+^), T_EM_ (CD45RO^+^CCR7^–^), T_EMRA_ (CD45RO^–^CCR7^–^). Cells with ambiguous phenotypes were excluded. (**C**) Distribution of phenotypes in **B** by clonotype frequency, ranked from largest to unique clonotypes. (**D**) The frequencies of YF45-specific T cells before vaccination and at the indicated days following YFV immunization. (**E**) Each circos plot represents TCRs from YF45 tet^+^ cells obtained 210–242 days after vaccination, separated by the associated indexed phenotypes. Cells are ordered by frequency within each arc. Gray marks cells expressing unique TCRs; other colors represent expanded or shared sequences. Shared TCRβ or TCRα and β, when a TCRα is available, is connected by a line across distinct phenotypic subsets. Blue lines highlight TCRs from T_MN_ cells that are shared with cells expressing other phenotypes. (**F**) The percentage of TCRs in each memory subset that matched T_MN_-derived sequences. T_EM_, effector memory; T_EMRA_, effector memory cells reexpressing CD45RA.

Although T_MN_ cells respond well to antigens in vitro, it remains possible for them to be less competitive in resource-limiting environments. To investigate this, we reason that we can use TCR sequences to infer stimulation and proliferative response in vivo. Because T cell progenies originating from a T cell express identical TCR sequences, we can further leverage these sequences as molecular barcodes to investigate the clonal relationship between distinct phenotypic subsets. However, capturing sufficient numbers of T_MN_ cells was challenging because of their limited number within the available blood samples. To overcome this problem, we generated new tetramers using affinity-matured HLA-DR monomers containing mutations that enhanced CD4 binding to improve the overall capture efficiency (24). When compared with the wild-type (WT) DR, these tetramers stained a larger population of T cells without significantly skewing the phenotypic proportions (Supplemental Figure 4, A–C). In total, we sorted single cells from 5 tetramer-labeled populations and obtained TCR sequences from 607 YFV-specific CD4^+^ T cells after amplification and sequencing (Figure 4A and Supplemental Table 4). Consistent with clonal expansion after vaccination, over 70% of the sequences were identified in multiple tetramer-sorted T cells. Among expanded sequences, 25% to 52% were abundant and found in at least 10 individual T cells (Figure 4B). Most T cells displayed a T_CM_ or T_EM_ phenotype based on antibody staining at the time of sorting. T_MN_ phenotype was infrequent, expressed by 3% to 4% of sequenced T cells and confined to the 2 most extensively sequenced populations recognizing YF45. Consistent with in vivo expansion, T_MN_ cells did not preferentially express unique TCRs, but rather, they were distributed across various clone sizes (Figure 4C). We focused the subsequent analyses on YF45-specific T cells that included the T_MN_ subset. Early post-vaccine measurements of YF45-specific T cells from HD2 and HD3 showed that both populations had generated robust responses to the YFV vaccine (Figure 4D) (21). In agreement with an antigen-driven response, T_MN_ cells contained expanded clonotypes and shared overlapping sequences with various memory subsets (Figure 4, E and F, and Supplemental Figure 4E). In separately generated T cell clones from the same individuals, T_MN_ cell–derived clones expressed TCRs that matched the sequences from ex vivo–sorted T cells of diverse clone sizes and phenotypes (Supplemental Figure 4F).

**Figure 5:**
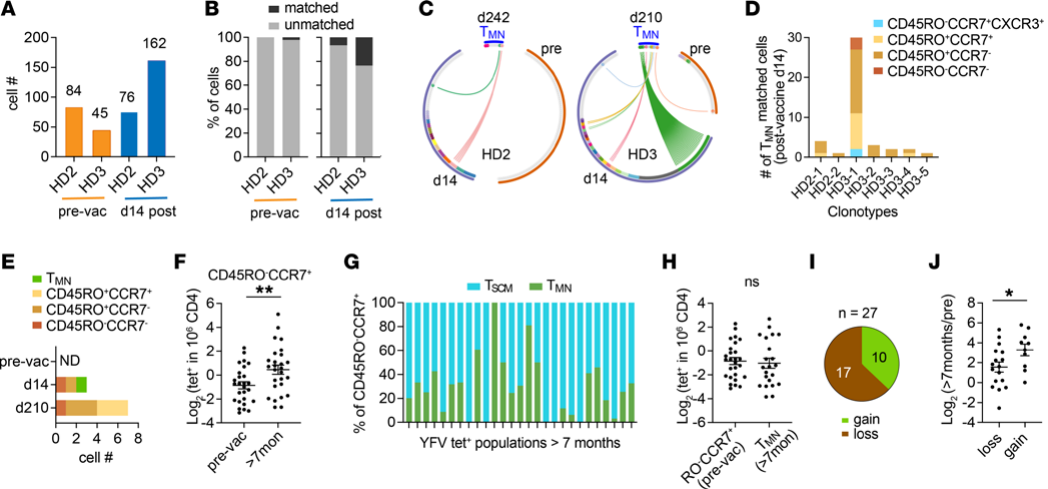
T_MN_ cells are clonally related to effector T cells and contribute to the maintenance of the naive repertoire after vaccination. (**A**) The number of TCRs from YF45 tet^+^ T cells from the indicated donors, before and 14 days after YFV vaccination. (**B**) TCRs from cells in **A** were compared to T_MN_-derived TCRs in the day 210–242 post-vaccine samples. Bar graphs show the percentages of cells with or without a match. (**C**) Each circos plot represents TCRs from T_MN_ YF45 tet^+^ cells and TCRs from the same specificities, before and 14 days after YFV vaccination, from the same donors. Cells are ordered by frequency within each arc. Gray marks cells expressing unique TCRs; other colors represent expanded or shared sequences. Connecting lines highlight shared TCRs between a T_MN_ cell and cells in a previous time point. (**D**) The number and phenotypes of T cells in the day 14 sample that matched a T_MN_-derived clonotype from the memory time point. (**E**) Clonal dynamics of a TCR expressed by a T_MN_ cell in the 14-day post-vaccine sample. Plot shows the number and phenotypes of T cells that expressed the same TCR sequence at the indicated time points. ND, not detected. (**F** and **G**) The frequencies of CD45RO^–^CCR7^+^ YFV-specific T cells before and after vaccination for the corresponding populations (**F**). Post-vaccine CD45RO^–^CCR7^+^tet^+^ cells were separated into T_SCM_ or T_MN_ subsets based on CXCR3, CD95, CD11a, or CD49d staining (**G**). (**H**) Plot compares the frequencies of CD45RO^–^CCR7^+^tet^+^ cells before vaccination with the frequencies of T_MN_ cells in the corresponding population from the post-vaccine sample. (**I**) The post-vaccine T_MN_ frequency of each tet^+^ population was divided by its initial CD45RO^–^CCR7^+^ frequency before vaccination. The ratio is defined as a gain if above 1 and a loss if below 1. Pie chart shows the numbers of populations that had gained or lost naive T cells after vaccination. (**J**) Plot shows the fold-change in tet^+^ frequency, from the pre-vaccine baseline to the memory time point, for populations that had gained or lost naive cells. For **F**, **H**, and **J**, *n* = 27 tetramer^+^ populations. Welch’s *t* test was performed. *P < 0.05, **P < 0.01.

The presence of shared TCR sequences with memory T cells, together with clonal expansion, suggest that T_MN_ cells had encountered and responded to antigens. Alternatively, clonotype sharing between memory and naive cells may be explained by the presence of multiple naive T cells with the same TCR, where a portion did not encounter YFV antigen and remained naive. To investigate this possibility, we examined the prevaccination repertoire of YF45-specific T cells in these individuals to determine if preexposure T cells expressing T_MN_-associated TCRs were abundant before vaccination (21). The changes in clonal dynamics were assessed by tetramer staining, sorting, and sequencing of the TCRs of YF45-specific T cells from blood collected 14 days after vaccination. Of note, the sampling depth at day 14 was much shallower because of limited sample availability. Ten million to 20 million PBMCs were used for tetramer staining whereas over 100 million CD4^+^ T cells were used to capture rare precursor T cells in the prevaccination sample. In total, we examined TCR sequences from 129 precursor T cells and 238 effector T cells (Figure 5A and Supplemental Table 5). Before vaccination, no pre-vaccine TCRs matched T_MN_-derived TCRs from HD2, and only 1 sequence was identified in HD3. This shared TCR mapped to a unique sequence and not to the expanded preexisting clonotypes in this individual (Figure 5, B and C). Fourteen days following vaccination, matched TCR frequencies increased to 7% in HD2 and 23% in HD3, capturing a total of 7 clonotypes (Figure 5, B and C). The majority of matched clonotypes were expanded and expressed by a variety of differentiated cells (Figure 5D). Among T cells without a T_MN_ match, 2 cells in HD3 were T_MN_ like and lacked differentiation marker expression. One of these cells was found as a part of an expanded clone at day 14 and shared the same TCR with multiple memory T cells in the day 210 sample (Figure 5E and Supplemental Table 5). Additionally, the post-vaccine day 14 blood from this donor contained 3 CD45RO^–^CCR7^+^ T cells without additional differentiation marker staining (Supplemental Table 5).

Human T cells stimulated with cytokines can maintain a naive phenotype (25). Our data suggest that this can also occur with antigen-specific responses. To broaden the analyses, we compared the frequency of CD45RO^–^CCR7^+^ T cells in various YFV-specific populations before and after YFV vaccination (Figure 5F). This showed an increase in CD45RO^–^CCR7^+^tetramer^+^ cells after exposure, which included diverse ratios of T_SCM_ and T_MN_ cells (Figure 5G). We focused on the T_MN_ subset as they resembled unstimulated naive T cells. If there is no mechanism to replenish the naive repertoire or hold on to a naive phenotype, we expected a decrease in naive cell frequency following an immune response as responding T cells acquired differentiated states. However, contrary to this, the averaged T_MN_ cell frequency after vaccination was comparable to the frequency of naive cells before vaccination (Figure 5H). To examine population-level differences, we divided the post-vaccine T_MN_ frequency of each population by its initial naive T cell frequency before vaccination. This revealed lower T_MN_ frequencies in two-thirds of the populations, while the remaining third showed an increase (Figure 5I). The absence of a reduction in naive T cells did not indicate a lack of responsiveness to the vaccine. Instead, among the 10 populations that gained naive T cells, there was a higher average fold-change in total tetramer^+^ frequency before and after vaccination (Figure 5J). Collectively, these data suggest the T_MN_ subset comprises T cells that had responded to vaccination.

### T_MN_ cells contribute to durable memory.

**Figure 6:**
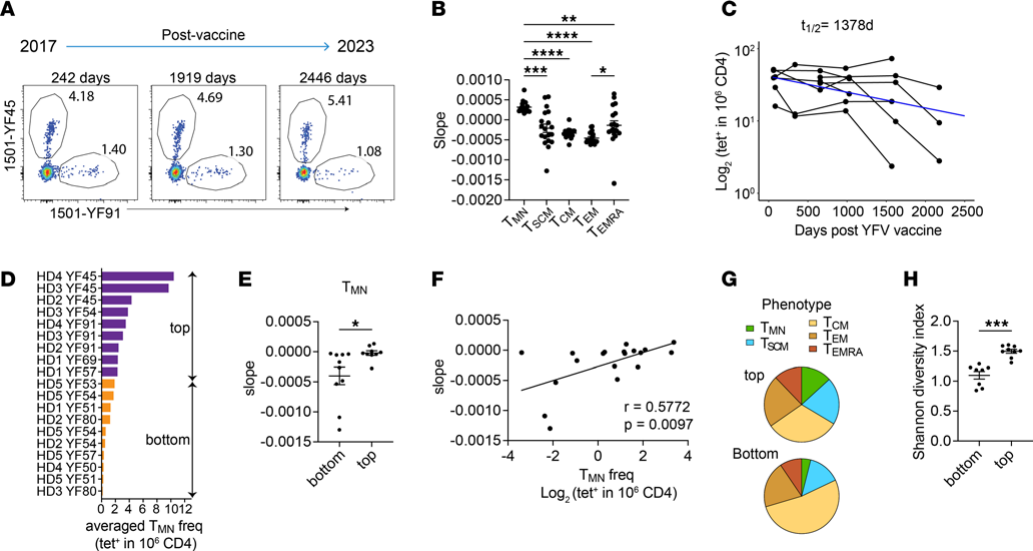
T_MN_ cells are stable and associated with durable T cell memory. (**A**) Representative plots show YFV-specific CD4^+^ T cells over the indicated time points from HD2. (**B**) Each tet^+^ population of a given specificity was subdivided according to phenotypes. The change over time for each phenotypic subset was quantified by the estimated slope using a mixed effects exponential decay model (*n* = 19 tetramer^+^ populations from 5 donors). (**C**) A mixed effects exponential decay model fitted to the dynamics of YFV-specific CD4^+^ T cells after a single YFV vaccination (*n* = 8 populations, combined from donors 4 and 5). The estimated decay (blue line) was used for calculating the *t_1/2_*. (**D**) Ranking of tet^+^ populations by the averaged frequency of T_MN_ cells within each population across all time points. (**E**) Plot summarizes the estimated slopes of individual tet^+^ populations, divided into top and bottom halves by T_MN_ frequency in **D**. (**F**) The correlation between slopes characterizing the change over time for the overall tetramer^+^ populations and their corresponding averaged T_MN_ frequencies. (**G**) Pie charts show the distribution of memory subsets. Populations were divided into top and bottom groups by the first measured T_MN_ frequency obtained within 1–2 years after YFV vaccination. (**H**) Phenotypic diversity of each tet^+^ population was quantified using Shannon diversity index, categorized into top or bottom groups based on T_MN_ frequency as in **G**. Each symbol represents 1 tetramer^+^ population. Experiments were repeated an average of 2.3 times. Data are represented as mean ± SEM. (**B**) RM 1-way ANOVA was performed and corrected with Tukey’s multiple comparisons test. (**E** and **H**) Welch’s *t* test was performed. (**F**) Spearman’s correlation was performed. *P < 0.05, **P < 0.01, ***P < 0.001, ****P < 0.0001.

While memory T cells are essential for generating rapid recall responses, naive T cells are known for their long lifespan and regenerative potential (26, 27). Therefore, we hypothesized that T_MN_ cells promote the durability of antigen-specific responses after vaccination. To test this idea, we analyzed additional time points from 5 donors with longitudinal PBMCs collected up to 6.7 years after YFV vaccination (Figure 6A and Supplemental Figure 5A). Past modeling of cellular turnover suggests that different phenotypic subpopulations undergo separate and distinct in vivo dynamics (28, 29). To evaluate the stability of individual phenotypic subsets, we subdivided 19 YFV-specific populations according to T_MN_, T_SCM_, T_CM_, T_EM_, and T_EMRA_ phenotypes based on CD45RO, CCR7, CD95, CXCR3, CD11a, and CD49d expression. Their time-dependent change was quantified as a fitted slope using a mixed effects exponential decay model. This revealed different rates of decay between cells in distinct differentiation states. CD4^+^ T_EM_ cells had the largest negative slope, indicating the greatest decrease over time. In contrast, T_MN_ cells exhibited remarkable stability, with no discernible decline observed during the follow-up period. The stability of the T_MN_ subset significantly surpassed that of other phenotypic subsets, including T_SCM_ and T_CM_ cells, which are typically considered long-lived (Figure 6B).

Next, we examined the decay kinetics of the overall tetramer^+^ populations. Because some data were generated before switching to modified DR, paired analyses by WT and modified tetramers on the same blood sample were used to generate an equation for normalizing the frequencies across experiments (Supplemental Figure 4D). Among the 5 donors followed longitudinally, 2 received 1 YFV dose as typical for the YFV vaccine, whereas 3 received a second YFV vaccine 7 months to a year after the initial dose (Supplemental Table 6). Revaccination was originally a part of an IRB-approved protocol to study recall response. Although this strategy did not effectively induce acute T cell responses, likely due to YFV neutralization by antibodies generated from the first vaccination, we investigated whether it might still affect long-term T cell dynamics. We divided tetramer^+^ populations based on donors’ vaccine doses. Consistent with the longevity of YFV vaccine–mediated protection, YFV-specific CD4^+^ T cells displayed an average half-life (*t_1/2_*) of close to 4 years after 1 YFV immunization (Figure 6C). This durable response was further stabilized after reexposure in the 2-dose group (Supplemental Figure 5B).

T_MN_ cell frequencies did not significantly differ between the 1-dose and 2-dose groups (Supplemental Figure 5C). Given the wide variation in T_MN_ cells, we divided tetramer^+^ populations based on T_MN_ frequency into top and bottom halves (Figure 6D). This showed that populations with more T_MN_ cells were more stable compared with populations in the bottom T_MN_ group (Figure 6E). The T_MN_ frequency within a given virus-specific population positively correlated with the stability of the overall population (Figure 6F). By contrast, we did not find significant differences between high and low groups based on T_SCM_, T_CM_, T_EM_, and T_EMRA_ frequencies (Supplemental Figure 5D). On the phenotypic level, all tetramer^+^ populations contained various memory subsets, but the top T_MN_ group was more phenotypically diverse. We divided populations based on the first T_MN_ frequency obtained within the 1–2 years after YFV vaccination and showed that populations with more T_MN_ cells exhibited greater diversity of differentiation states over time as measured by the Shannon diversity index (Figure 6, G and H). Collectively, these data highlight the stability of the T_MN_ subset and uncover their association with durable and diverse T cell memory after YFV vaccination.

## Discussion

A T cell is typically referred to as naive if it has not yet encountered its specific cognate antigen(s). However, a clear antigenic history is often not available in human studies, and thus specific surface markers are commonly used to infer antigen experience (11, 30). Here, we examined CD4^+^ T cell memory to YFV vaccination to define key features of durable memory by direct ex vivo class II tetramer staining and enrichment. This showed a diverse memory pool composed of various differentiation states after YFV vaccination. Surprisingly, we also uncovered an increase in naive-phenotype YFV-specific T cells after vaccination.

Deeper phenotypic analyses showed that a portion of CD45RO^–^CCR7^+^ cells were previously described T_SCM_ cells. The remaining cells did not express known surface markers of T cell differentiation that were tested. We evaluated several potential explanations for their origin. Our initial idea that T_MN_ cells were unresponsive was incorrect. Peptide-stimulated T_MN_ cell–derived T cell clones produced robust levels of cytokines at comparable EC_50_ as T cell clones generated from memory cells. The presence of expanded TCR clones within the T_MN_ subset further suggests that some T_MN_ cells had encountered and responded to antigens in vivo. The observation that T_MN_ cells, memory T cells, and effector T cells have overlapping TCR repertoires suggests shared precursors among these subsets and provides additional support to the idea that some T_MN_ cells are antigen experienced, as their common precursors divided at least once in response to antigen. This extent of overlap would not be expected if clonal expansion within the T_MN_ subset were driven by different processes, such as cytokine-mediated and/or bystander response. Our interpretation that some expanded antigen-experienced cells exist within the T_MN_ population is further supported by increased naive T cell frequency following immunization. We show that, despite recruitment into effector responses, the frequency of naive T cell frequency recognizing YFV is largely unchanged. Among populations analyzed before and after immunization, approximately one-third gained more naive-phenotype T cells in the form of T_MN_ cells above their pre-vaccine baseline. It is also unlikely we would capture T_MN_ cells 14 days after vaccination if they had not expanded, since the same clonotype was not detected before vaccination despite analyzing many more cells.

In addition to an antigen-experienced subset, some T_MN_ cells could be genuine naive T cells. As an alternative possibility, there might be multiple naive cells of the same clonotype, but only a subset responded to the vaccine. Incomplete response may have occurred because some YFV-specific T cells were not at the right place or time to respond to the immunogen. Other cells may not have received enough of the appropriate signal to undergo expansion and/or differentiation (31). This model could be consistent with our TCR data but does not explain why certain specificities contain higher frequencies of naive T cells after vaccination. An intriguing alternative for this increase might involve ongoing thymic activities. Although thymic output for new T cells declines with age, recent thymic emigrants remain detectable in adults (32–34). Newly produced T cells might add to the peripheral T_MN_ pool to increase the abundance of YFV-specific naive T cells after vaccination. T_MN_ cells from various sources could contribute to the naive repertoire after antigen exposures, varying in their impact depending on antigen specificity, the type of stimulation, age, and other individual characteristics.

Only a few select vaccines are capable of mediating lifelong protection. How durable immunological memory is maintained remains a key unresolved question. While memory T cells are the cornerstone of protective immunity by virtue of their ability to rapidly initiate a functional response to pathogen rechallenge, naive T cells possess superior self-renewal capacity and differentiation plasticity (3, 26, 27, 35). Irrespective of how T_MN_ cells might have originated, we asked whether T_MN_ cells are related to the longevity of T cell response. Our findings revealed remarkable stability of the T_MN_ population, showing minimal decay for nearly 7 years. These stable T cells could potentially support the longevity of the overall immune response, extending it beyond the lifespan of individual memory T cells. Consistent with this model, T_MN_ cells are more abundant in durable CD4^+^ populations that are stable over time. Based on the diverse memory phenotypes in T_MN_-enriched populations, we further speculate that T_MN_ cells have the potential to differentiate into multiple states, thereby contributing to the phenotypic diversity of T cell memory.

A limitation of our study is that we did not further subset T_MN_ cells to evaluate potential heterogeneity in gene expression, epigenetic landscape, or functional attributes because of the limitations on cell numbers. Future studies using models allowing manipulation of T_MN_ cells will be needed to build on their association with durable immunity and establish causation. As our analyses are focused on CD4^+^ T cell responses to YFV in a few healthy individuals, larger studies involving additional vaccines will be important to strengthen our findings and to understand how the T_MN_ subset influences vaccine durability across different ages and disease settings.

In summary, our findings highlight the complexity within the post-immune T cell compartment and add to our understanding of the diverse spectrum of T cells exhibiting naive features (27, 36). Using an antigen-specific approach, our data suggest that some cells considered naive by phenotypic criteria are actually antigen experienced. As a whole, post-immune T_MN_ cells are remarkably stable. Understanding how T_MN_ cells are generated, are maintained, and work alongside memory cells in providing long-lasting immune protection could aid the future development of improved vaccine strategies.

## Methods

### Sex as a biological variable

Both male and female participants were included in this study. Study volunteers were recruited in the order of participation without restrictions based on the sex.

### Human samples

This study used cryopreserved cells stored in FBS with 10% DMSO from an ongoing vaccine study at the University of Pennsylvania (21). This study includes 7 healthy adult participants with no prior YFV exposure who received 1 or 2 doses of the 17D live-attenuated YFV vaccine (YF-VAX, Sanofi Pasteur). Individuals older than 65 were excluded. As YFV vaccine provides lifelong protection against YFV, revaccination with the attenuated 17D-204 strain is not expected to increase risk. Five participants were followed longitudinally for 2 to 6.7 years after vaccination. Participant characteristics are shown in Supplemental Table 1.

### Cell lines

Hi5 cells (Thermo Fisher Scientific) were maintained by insect cell culture medium (ESF921, Expression Systems) supplemented with 0.02% gentamicin at 28°C.

### Protein expression and tetramer production

His-tagged HLA-DRA/B1*0301, 0401, 0407, and 1501 protein monomers of WT sequence or with L112W, S118H, V143M, or T157I mutations (24) were produced by Hi5 insect cells and extracted from culture supernatant using Ni-NTA (QIAGEN). HLA-DR monomers were biotinylated overnight at 4°C using BirA biotin ligase (Avidity) and purified by size-exclusion chromatography using Superdex 200 size-exclusion column (AKTA, GE Healthcare, now Cytiva). Biotinylation was confirmed by gel-shift assay. Peptide exchange and tetramerization for WT and modified affinity-matured DR were performed using standard protocols as previously described (37, 38). In brief, HLA-DR proteins were incubated with thrombin (MilliporeSigma) at room temperature for 3–4 hours and exchanged with peptides of interest in 50-fold excess at 37°C for 16 hours. Peptide-loaded HLA-DR monomers were incubated with fluorochrome-conjugated streptavidin at 4–5:1 ratio for 2 minutes at room temperature, followed by a 15-minute incubation with an equal volume of biotin-agarose slurry (MilliporeSigma). Tetramers were buffer exchanged into PBS, concentrated using Amicon ULTRA 0.5 mL 100 kDa (MilliporeSigma), and kept at 4°C for no more than 2 weeks prior to use.

### Ex vivo T cell analyses and cell sorting

#### Phenotypic analyses and frequency quantification.

Tetramer staining was performed on at least 10,000,000 PBMCs with 5 μg of tetramers in a 100 μL reaction for 1 hour at room temperature as previously described (21, 38, 39). Tetramer-tagged cells were enriched by adding anti-fluorochrome and anti-HIS MicroBeads (Miltenyi Biotec). The mixture was passed through LS columns (Miltenyi Biotec). Column-bound cells were washed and eluted according to manufacturer protocol. For antibody staining, the enriched samples were stained with viability dyes and exclusion markers (anti-CD19 and anti-CD11b, Supplemental Table 7), along with combinations of surface markers as specified in the experiments (anti-CD3, anti-CD4, anti-CD45RO, anti-CCR7, anti-CD11a, anti-CD95, anti-CD49d, and anti-CXCR3, Supplemental Table 7), in 50 to 100 μL of FACS buffer (PBS plus 2% FCS, 2.5 mM EDTA, 0.025% sodium azide) for 30 minutes at 4°C. Samples were fixed with 2% paraformaldehyde and acquired by flow cytometry using LSRII (BD). Data analyses were performed by FlowJo (BD). The Boolean tool was used to define T_SCM_ and T_MN_ cells within the CD45RO^–^CCR7^+^ subset. Frequency calculation was obtained by mixing one-tenth of samples with 200,000 fluorescence beads (Spherotech) for normalization.

For longitudinal experiments involving both WT and modified DR, paired data from WT and modified DR, with a minimum of 2 data points per time point for each specificity, were used to derive the equation for normalization: log_2_(Freq_modified_) = 3.72 + 0.35 × log_2_(Freq_WT_) (Supplemental Figure 4D). Frequencies generated by WT tetramers that were below the normalized values were adjusted. Mixed effects exponential decay models were used to analyze longitudinal changes in antigen-specific T cell populations and estimate the corresponding slopes. These models were implemented in MonolixSuite 2021R1 (Lixoft) and fitted to data after vaccination. Initial T cell specificity values were lognormally distributed, exponential decay rates were normally distributed, and lognormal multiplicative error was used. The estimation of the population parameters was performed using the Stochastic Approximation Expectation-Maximization algorithm. Half-lives were calculated as ln(2)/*k*, where the corresponding *k* values represented the estimated exponential decay rate constants. Estimated decay rates were converted into slopes as –*k*.

For multidimensional analyses, a total of 1,465 manually gated tetramer^+^ cells were exported from FlowJo, read into R by flowCore, and combined into 1 data set for subsequent data processing and analyses using the Spectre package in R (22). Staining intensities were converted using Arcsinh transformation with a cofactor of 200. Batch alignment was performed using CytoNorm (40). Clustering was performed using Phenograph with nearest neighbors set to 55 (*k* = 55) (41). UMAP was used for dimensional reduction and visualization (42).

#### Function response.

T cells were rested overnight, followed by 4–5 hours of stimulation by PMA (5 ng/mL, MilliporeSigma) and ionomycin (500 ng/mL, MilliporeSigma) in the presence of monensin (2 μM, MilliporeSigma) and Brefeldin A (5 μg/mL, MilliporeSigma). Tetramer and surface antibody staining were performed as above. Intracellular staining with antibodies against TNF-α, IFN-γ, CD3, and CD4 (Supplemental Table 7) was performed following BD Cytofix/Cytoperm Fixation/Permeabilization Kit according to manufacturer protocol.

#### Cell sorting.

Around 60,000,000 CD3^+^ or CD4^+^ T cells were used and stained with up to 10 μg of each tetramer in a 100 μL reaction. Antibody staining was performed as above without fixation. Individual tetramer-labeled cells were isolated for TCR sequencing or T cell cloning by index sorting using the purity mode on FACSAria (BD).

### Generation and stimulation of T cell clones

#### Clone generation.

Cells were stained with tetramers and enriched with magnetic beads as described above. Single-tetramer-stained CD4^+^ T cells were sorted into individual wells in a round-bottom, 96-well plate containing 10^5^ irradiated PBMCs, 10^4^ JY cell line (Thermo Fisher Scientific), PHA (1:100, Thermo Fisher Scientific), IL-7 (25 ng/mL, PeproTech), and IL-15 (25 ng/mL, PeproTech). IL-2 (50 IU/mL, PeproTech) was added on day 5 and replenished every 3–5 days. Cells were resupplied with fresh medium with IL-2 (50 IU/mL), PHA (1:100), and 10^5^ irradiated PBMCs every 2 weeks.

#### DC generation.

Monocytes from HLA-DR allele-matched donors were isolated using negative enrichment kits (RosetteSep Human Monocyte Enrichment Cocktail, StemCell Technology). A total of 10,000,000 cryopreserved monocytes were cultured in 15 mL DC media (RPMI 1640 plus glutamine, 10% FCS, 1× penicillin/streptomycin, 10 mM HEPES) in the presence of 100 ng/mL GM-CSF and 500 U/mL IL-4. Three days later, half the culture media was replaced with fresh DC media with 100 ng/mL GM-CSF, 500 U/mL IL-4, and 0.05 mM 2-mercaptoethanol. Cells in suspension were harvested at 5 to 6 days and added to a flat-bottom, 96-well plate at 25,000 DCs per well. DCs were treated with 100 ng LPS and peptides (0.00001 μg/mL to 10 μg/mL) for 16 hours and replenished with fresh media before coculturing with T cells.

#### Stimulation of T cell clones.

T cell clones were rested overnight in fresh media without IL-2 and added to wells containing matured DCs at 1:1 ratio in the presence of monensin (2 μM, MilliporeSigma) and Brefeldin A (5 μg/mL, MilliporeSigma). After 5 hours, cells were transferred into a new 96-well, round-bottom plate, washed once with FACS buffer, and stained with viability dyes and exclusion markers (anti-CD19 and anti-CD11b) for 30 minutes at 4°C. Intracellular staining with antibodies against TNF-α, IFN-γ, IL-2, CD3, and CD4 (Supplemental Table 7) was performed following BD Cytofix/Cytoperm Fixation/Permeabilization Kit according to manufacturer protocol. EC_50_ was determined using the percentage of T cell clones that produced TNF-α in response to decreasing peptide concentrations (10, 1, 0.1, 0.01, 0.001, 0.0001, and 0.00001 μg/mL). A nonlinear fit without constraint was applied to log-transformed concentration using the equation Y = Bottom + (Top – Bottom)/(1 + 10^[(LogEC50^^–^^X)^^×^^HillSlope]^) in Prism (GraphPad). For the proliferation assay, T cell clones were labeled with 1:1,000 diluted CTV (Thermo Fisher Scientific) following manufacturer protocol. The CTV-stained cells were rested in fresh media without IL-2 for 16 hours. A total of 25,000 rested T cells were cocultured with DCs pulsed with 10 μg/mL cognate peptides or treated with PHA as a positive control (1:100, Thermo Fisher Scientific). After 5 days, cells were harvested and stained with viability dyes and surface antibodies (anti-CD19, anti-CD11b, anti-CD3, and anti-CD4 for 30 minutes at 4°C followed by fixation with 2% paraformaldehyde. Samples were acquired by flow cytometry using LSRII and analyzed by FlowJo.

### Single-cell TCR sequencing and analyses

Single-cell TCR sequencing by nested PCRs was performed using the primer sets and the protocol as previously described (21, 43). In brief, reverse transcription was performed with CellsDirect One-Step qRT-PCR kit according to the manufacturer’s instructions (CellsDirect, Invitrogen) using a pool of 5′ TRVB region–specific primers and 3′ C region primers. The cDNA library was amplified using a second set of multiple internally nested V-region and C-region primers with HotStarTaq DNA polymerase kit (QIAGEN). The final PCR was performed on an aliquot of the second reaction using a primer containing common base sequence and a third internally nested Cβ primer. PCR products were gel purified (QIAGEN) and sequenced on a NovaSeq 6000 platform (Illumina). TCR sequences were pre-processed as previously described (21). In brief, forward and reverse reads were converted into 1 paired-end read using pandaseq (44). Data were demultiplexed by the unique combination of plate, row, and column barcodes. Consensus TCRβ sequences were identified using the V(D)J alignment software MiXCR (45). A threshold of a read count of 200 reads per sequence was applied to the consensus sequences. If more than 1 TCRα or TCRβ chain passes this criterion, we retain the dominant TCRβ and the 2 TCRα chains with the highest read count. For data obtained from cells several months after vaccination, we additionally require phenotypic annotation based on antibody staining from index sort data. Data were excluded if phenotypic information was not retained or was ambiguous. For downstream analyses, data wrangling was performed using the tidyverse package. TCRs were matched by TCRβ if only the β chain was available or by TCRβ plus at least 1 TCRα if α chain(s) were called. Circos plots were made using the circlize package of R software (46).

### Statistics

Normality was assessed using D’Agostino-Pearson test. Spearman’s was used if either of the 2 variables being correlated was non-normal. Otherwise, Pearson’s was used to measure the degree of association. Least squares linear regression was used to calculate the best fitting line. Statistical comparisons were performed using 2-tailed Student’s *t* test, paired *t* test, Welch’s 1-way ANOVA, repeated measures 1-way ANOVA, 2-way ANOVA, or mixed effects model. A *P* value of less than 0.05 was used as the significance level and adjusted using Tukey’s multiple comparisons test or Dunn’s multiple comparison test, as indicated in the figure legends, if multiple comparisons were performed. Statistical analyses were performed using GraphPad Prism. Lines and bars represent the mean, and variability is represented by the SEM.

### Study approval

All participants have given written informed consent. All samples were deidentified and obtained with IRB regulatory approval from the University of Pennsylvania. The study was approved by the IRB at the University of Pennsylvania (approval 820884).

### Data availability

All data needed to evaluate the conclusions in the paper are present in the paper or the supplement. Analyses are performed using standard analysis packages. All data points are reported in the Supporting Data Values file.

## Author contributions

LFS was responsible for conceptualization, YP for experimentation, LB for sequence analyses, RX for high-dimensional phenotypic analyses, BVP for study recruitment, VIZ for modeling and statistical support, and LFS for supervision; LFS, VIZ, LB, and YP were responsible for manuscript preparation.

## Supplementary Material

Supplemental data

Supporting data values

## Version 1 06/11/2024

In-Press Preview

## Version 2 07/22/2024

Electronic publication
