# Supplementary material for: Preservation of naive-phenotype CD4+ T cells after vaccination contributes to durable immunity
Source: JCI Insight. 2024 Jun 11;9(14):e180667. doi: 10.1172/jci.insight.180667 (PMC11383171; doi:10.1172/jci.insight.180667)
Supplement: Supplemental data [file jciinsight-9-180667-s017.pdf]

Supplementary Material

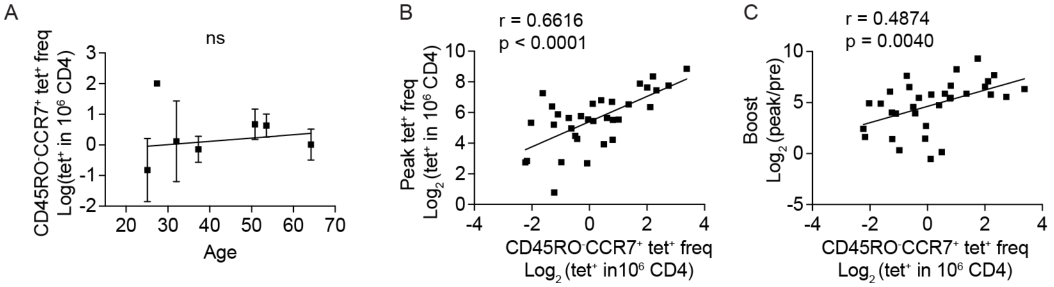

Figure S1: CD45RO$^-$CCR7$^+$ YFV-specific CD4$^+$ T cells by age and their relationship to the effector response. (A) The frequency of post-immune CD45RO$^-$CCR7$^+$ YFV tet$^+$ CD4$^+$ T cells in relationship to donor age. Distinct tet$^+$ populations from the same donor are combined and represented as an average (n = 7). (B) The correlation between the frequency of CD45RO$^-$CCR7$^+$ YFV tet$^+$ cells measured at least 7 months after vaccination and the highest total tet$^+$ frequency from a previous time point measured within the first month after vaccination (n = 36). (C) The correlation between CD45RO$^-$CCR7$^+$ YFV tet$^+$ T cell frequency at least 7 months after vaccination and the fold-change between peak frequency and the pre-vaccine baseline. Pearson correlation was computed.

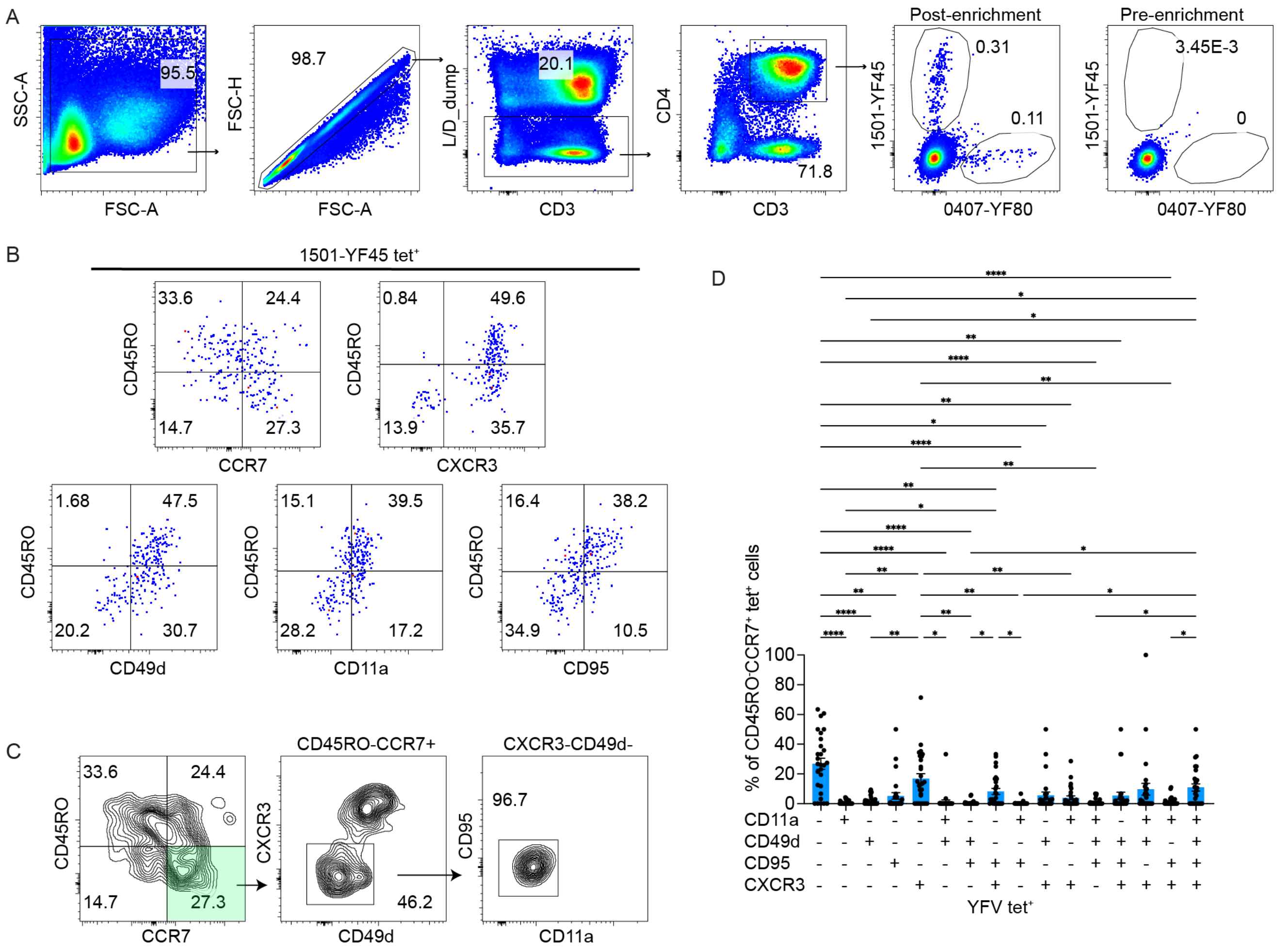

Figure S2: YFV tetramer^{+} CD45RO^{-}CCR7^{+} T cells contain various phenotypic subsets. (A) Representative plots show the gating strategy used to identify tetramer^{+} cells. (B) FACS plots show the phenotype of YF45-specific T cells by the indicated antibody staining. (C) Representative plots show manual gating for YF45-specific T_{MN} cells from HD3. (D) Boolean gates for CD11a, CD49d, CD95, and CXCR3 were applied onto manually gated CD45RO^{-}CCR7^{+} YFV tet^{+} T cells. The plot shows various phenotypic combinations. Each symbol represents a tetramer^{+} population (n = 28). One-way ANOVA was performed and corrected with Tukey's multiple comparison test.

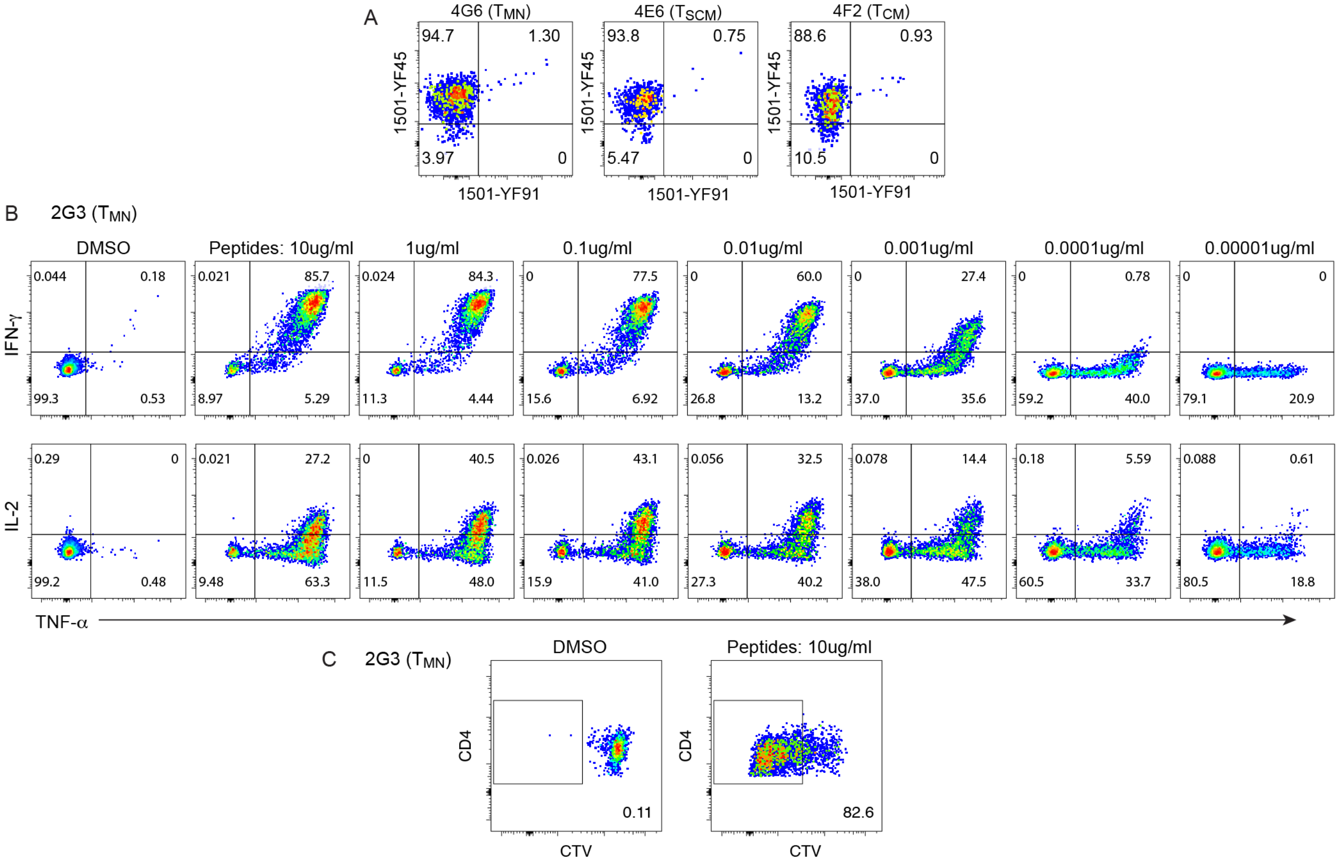

Figure S3: Tetramer staining and peptide responses of YFV-specific T cell clones. (A) Representative tetramer staining of T_{MN}, T_{SCM}, and T_{CM}-derived YFV-specific T clones. (B) Plots show cytokine response by a T_{MN} clone to decreasing concentrations of the cognate YF45 peptides after a 5-hour co-culture with peptide-loaded DCs. (C) Representative plots show CTV staining of a CTV-labeled T cell clone after a 5-day culture with vehicle-treated or peptide-loaded DCs.

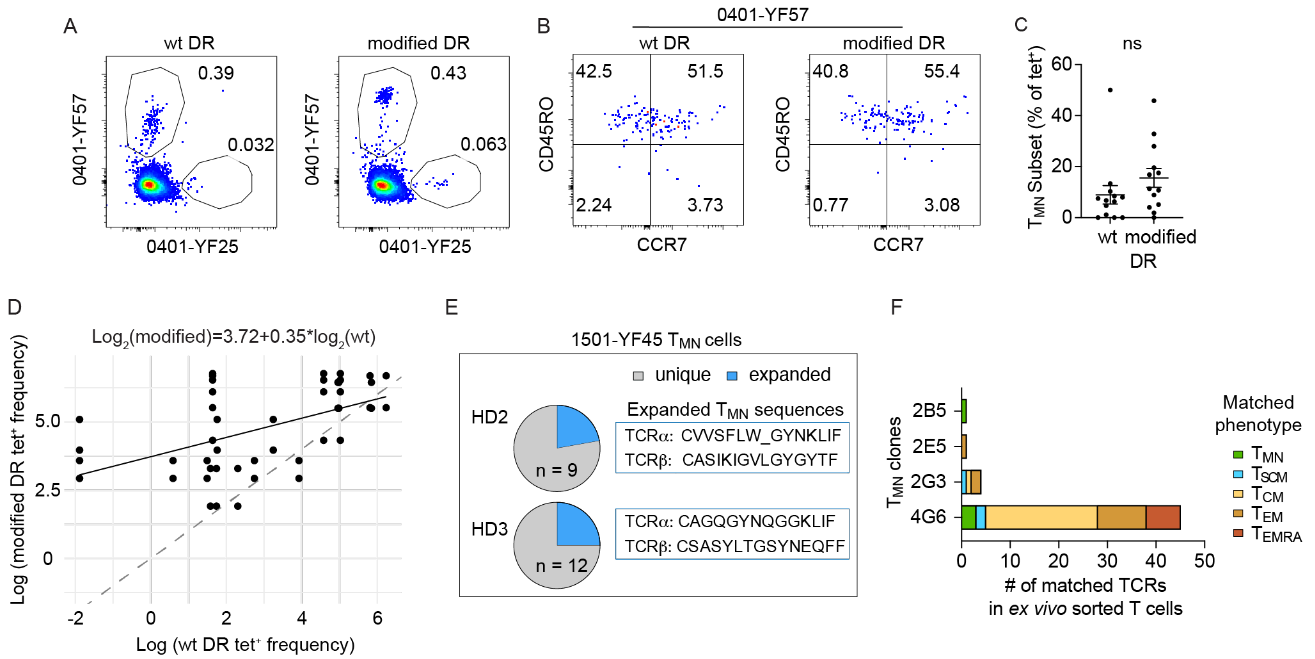

Figure S4: T_{MN} cells are antigen-experienced. (A) Representative plots show the identification of YFV-specific T cells using tetramers generated from wt or modified DR (L112W, S118H, V143M, T157I). (B) CD45RO and CCR7 expression of YF57-specific T cells stained by wt or modified DR. (C) Quantification of T_{MN} fraction within tet$^+$ populations identified by wt or modified tetramers using the same sample. (D) The plot displays paired data from wt and modified DR, with a minimum of two data points per time point for each specificity, which were used to derive the equation for normalization (top). (E) Pie-charts show the distribution of unique versus expanded clonotypes within T_{MN} subset of YF45-specific T cells obtained 210-242 days after YFV vaccination. The sequences of the expanded clonotype from each donor are as indicated. (F) T_{MN}-derived T cell clones were re-stained with tetramers and sorted for TCR sequencing. Clone-derived TCRs were compared with sequences from directly sorted T cells. The graph shows T_{MN}-derived clones and the number of matched TCRs from *ex vivo* sorted T cells with their phenotypes. For (C), Wilcoxon matched-pairs signed rank test was performed.

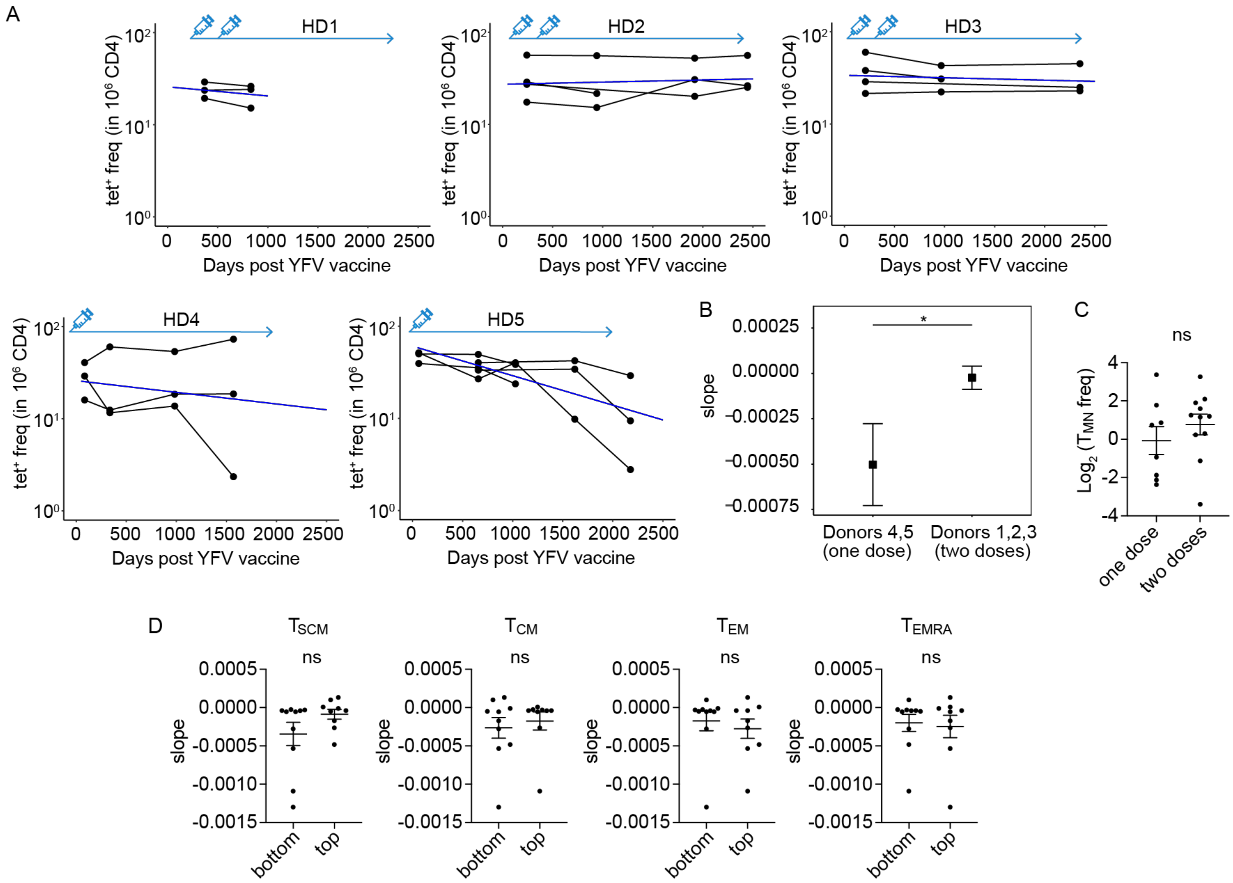

Figure S5: Longitudinal dynamics of YFV-specific populations. (A) The frequency of tetramer$^+$ populations by donor. Filled circles represent individual tetramer$^+$ populations. A line connects T cells labeled by the same tetramer across time points. Mixed effects exponential decay models were fitted to the longitudinal frequencies of individual tetramer-labeled populations for each donor. Blue lines represent the estimated decay. (B) Longitudinal dynamics of tetramer$^+$ populations were combined for donors who received one YFV dose (donors 4 and 5) and two doses (donors 1, 2, 3). A mixed-effects exponential decay model was employed to estimate the corresponding population slopes for the two groups. (C) The plot summarizes T_{MN} frequency in donors who received one (donors 4, 5) or two doses (donors 1, 2, 3) of the YFV vaccine. (D) Plots summarize the estimated slopes of individual tetramer$^+$ populations, divided into top and bottom halves by the averaged frequency of cells expressing the indicated phenotypes. (B) Wald test was used. (C) Mann-Whitney test was used. (D) Welch's t-test was used.

Table S1: Donor characteristics

ID	Sex	Age at the time of YFV vaccination	Vaccine dates	Sample collection dates
HD1	M	37	8/25/16	8/28/17
HD2	F	32	9/12/16	5/12/17
HD3	M	64	1/4/17	8/2/17
HD4	M	25	5/22/17	4/23/18
HD5	F	50	4/21/17	2/7/19
HD6	M	53	3/6/17	10/24/19
HD7	M	27	3/6/17	2/23/21

Table S2: List of YFV peptides

Peptide	YFV Protein	Sequence
YF108	NS5	VIKILTYPWDRIEEVTR
YF23	NS3	ESATILMTATPPGTS
YF25	NS3	KGPLRISASSAAQRR
YF38	RNA Polymerase	EEFIAKVRSHAAIGA
YF42	RNA Polymerase	ACLSKAYANMWSLMY
YF44	NS5	IHLVIHRIRTLIGQE
YF45	NS3	GEVIGLYGNGILVGD
YF50	Protein E	TIRVLALGNQEGSLKTA
YF51	Protein E	TDKMFFVKNPTDTGHGT
YF53	NS3	GLYGNGILVGDNSFVSA
YF54	NS3	LPSIRAANVMAASLRKA
YF57	NS5	TLGEVWKRELNLLDKRQ
YF69	NS3	WILADKRPTAWFLPSIR
YF80	Protein E	TGHGTVVMQVKVSKGAP
YF91	NS5	PPAGTRKIMKVVNRWLF
YF93	Protein E	MGAVLIWVGINTRNMTM

Table S3: Phenotypic markers

Phenotype	CD45RO and CCR7 staining	CD95, CXCR3, CD49d, CD11a staining
Naive	CD45RO-CCR7+	Staining not performed
T_{CM}	CD45RO+CCR7+	Not applicable
T_{EM}	CD45RO+CCR7-	Not applicable
T_{EMRA}	CD45RO-CCR7-	Not applicable
T_{SCM}	CD45RO-CCR7+	Positive for one of the following: CD95, CXCR3, CD49d, CD11a
T_{MN}	CD45RO-CCR7+	Negative for all of the following: CD95, CXCR3, CD49d, CD11a
NA	Staining not performed	Staining not performed

Table S4: YFV-specific CD4$^+$ T cells 7months or longer after vaccination
*See Table S3 for markers used to define each phenotype

Donor	specificity	Phenotype*	CDR	Clone size
HD2	0407-YF80	Naive	CASSSGGATNEKLFF	1
HD2	0407-YF80	Naive	CASRWDGNSPLHF	5
HD2	0407-YF80	TCM	CASIEGGEGDTQYF	1
HD2	0407-YF80	TCM	CASLTSGEGATEAFF	1
HD2	0407-YF80	TCM	CVVSSRHTDKLIF-CASPKSSGSTDTQYF	1
HD2	0407-YF80	TCM	CVVSSRHTDKLIF-CASRVTDNEQFF	1
HD2	0407-YF80	TCM	CALITQGGSEKLVF-CASRWDGNSPLHF;CVVSSRHTDKLIF-CASRWDGNSPLHF	1
HD2	0407-YF80	TCM	CASSLGSAGANVLTF	1
HD2	0407-YF80	TCM	CASSYRDRAFSRRGTEAFF	1
HD2	0407-YF80	TCM	CVVEGQTEAFF	1
HD2	0407-YF80	TCM	CSVEGTSGRGEQFF	2
HD2	0407-YF80	TCM	CSVEGTSGRGEQFF	2
HD2	0407-YF80	TCM	CASEGGGSGANVLTF	3
HD2	0407-YF80	TCM	CASIGGGEGNEQFF	5
HD2	0407-YF80	TCM	CASIGGGEGNEQFF	5
HD2	0407-YF80	TCM	CASIGGGEGNEQFF	5
HD2	0407-YF80	TCM	CASIGGGEGNEQFF	5
HD2	0407-YF80	TCM	CASRWDGNSPLHF	5
HD2	0407-YF80	TCM	CASRWDGNSPLHF	5
HD2	0407-YF80	TCM	CASRWDGNSPLHF	5
HD2	0407-YF80	TCM	CASGTGSSGANVLTF	8
HD2	0407-YF80	TCM	CASGTGSSGANVLTF	8
HD2	0407-YF80	TCM	CASGTGSSGANVLTF	8
HD2	0407-YF80	TCM	CASPKSSGSTDTQYF	12
HD2	0407-YF80	TCM	CASPKSSGSTDTQYF	12
HD2	0407-YF80	TEM	CVVSSRHTDKLIF-CASGTGSSGANVLTF;CALITQGGSEKLVF-CASGTGSSGANVLTF	1
HD2	0407-YF80	TEM	CALITQGGSEKLVF-CASPKSSGSTDTQYF	1
HD2	0407-YF80	TEM	CASSFSSGTAGPPLHF	1
HD2	0407-YF80	TEM	CALITQGGSEKLVF-CASSLGSAGANVLTF;CAPDNYGQNFVF-CASSLGSAGANVLTF	1
HD2	0407-YF80	TEM	CAVERQGSGNTGKLIF-CASSLGSAGANVLTF	1
HD2	0407-YF80	TEM	CVVSSRHTDKLIF-CASSLNPLGPWT_GD*GTNEKLFF	1
HD2	0407-YF80	TEM	CATNHGGSGANVLTF	1
HD2	0407-YF80	TEM	CATSSDRVREKLFF	1
HD2	0407-YF80	TEM	CASSLNTGVKQPQHF	2
HD2	0407-YF80	TEM	CASSLNTGVKQPQHF	2
HD2	0407-YF80	TEM	CASEGGGSGANVLTF	3
HD2	0407-YF80	TEM	CASEGGGSGANVLTF	3
HD2	0407-YF80	TEM	CASIGGGEGNEQFF	5
HD2	0407-YF80	TEM	CASRWDGNSPLHF	5
HD2	0407-YF80	TEM	CASGTGSSGANVLTF	8
HD2	0407-YF80	TEM	CASGTGSSGANVLTF	8
HD2	0407-YF80	TEM	CASGTGSSGANVLTF	8
HD2	0407-YF80	TEM	CASGTGSSGANVLTF	8
HD2	0407-YF80	TEM	CASGTGSSGANVLTF	8
HD2	0407-YF80	TEM	CASPKSSGSTDTQYF	12
HD2	0407-YF80	TEM	CASPKSSGSTDTQYF	12
HD2	0407-YF80	TEM	CASPKSSGSTDTQYF	12
HD2	0407-YF80	TEM	CASPKSSGSTDTQYF	12
HD2	0407-YF80	TEM	CASPKSSGSTDTQYF	12
HD2	0407-YF80	TEM	CASPKSSGSTDTQYF	12
HD2	0407-YF80	TEM	CASPKSSGSTDTQYF	12
HD2	0407-YF80	TEM	CASPKSSGSTDTQYF	12
HD2	0407-YF80	TEM	CASPKSSGSTDTQYF	12
HD2	0407-YF80	TEM	CASPKSSGSTDTQYF	12
HD2	1501-YF45	Naive	CALRGGGADGLTF-CASSPLSGGAYKTQYF	1
HD2	1501-YF45	Naive	CASSPSRPGGPLSYEQYF	1
HD2	1501-YF45	Naive	CIVRNSGNTPLVF-CSANRGAVSNQPQHF	1

HD2	1501-YF45	Naive	CGADMGGAGTASKLTF-CSANVATNSPLHF	1
HD2	1501-YF45	Naive	CATVDNQGGKLIF-CASSPSRPGGPLSYEQYF	2
HD2	1501-YF45	Naive	CAVLLRL_ATNKLIF-CSASARVGGELFF	2
HD2	1501-YF45	Naive	CIVRGNQFYF-CSASPRVEGEQYF	2
HD2	1501-YF45	Naive	CAMREGPYNFNKFYF-CASSLSRQGAREQFF	3
HD2	1501-YF45	Naive	CVVSVGKFSDGQKLLF-CASSQRQGINTEAFF	3
HD2	1501-YF45	Naive	CIVRVAAGNTGKLIF-CASQTGASVTYEQYF	9
HD2	1501-YF45	Naive	CIVRVAAGNTGKLIF-CASQTGASVTYEQYF	9
HD2	1501-YF45	Naive	CIVRVAAGNTGKLIF-CASQTGASVTYEQYF	9
HD2	1501-YF45	Naive	CIALPAGGTSYGKLTF-CASSPTGLGDYGYTF;CIALPAGGTSYGKLTF-CASSPTGLGDYGYTF	15
HD2	1501-YF45	Naive	CIALPAGGTSYGKLTF-CASSPTGLGDYGYTF	15
HD2	1501-YF45	Naive	CIALPAGGTSYGKLTF-CASSPTGLGDYGYTF	15
HD2	1501-YF45	TCM	CIVRENSGNTGKLIF-CASHEGWTVGNTIYF	1
HD2	1501-YF45	TCM	CIVRVTNQAGTALIF-CASSGKMTSFSYEQYF	1
HD2	1501-YF45	TCM	CARMSGGFKTIF-CASSLAGVGPGGYEQFF	1
HD2	1501-YF45	TCM	CAFMRGAGANNLFF-CASSLGLSVANEQFF	1
HD2	1501-YF45	TCM	CAVGAQGRGFQKLVF-CASSPGGLSTEAFF	1
HD2	1501-YF45	TCM	CASSPGGLSTEAFF	1
HD2	1501-YF45	TCM	CIVRALSGNTGKLIF-CASSPGQTLVTEAFF	1
HD2	1501-YF45	TCM	CIVRPSAGGGNKLTF-CASSSGEVLGEQYF	1
HD2	1501-YF45	TCM	CASSSGGLNTEAFF	1
HD2	1501-YF45	TCM	CIVRYSGGSNYKLTF-CASSSGSNTVNTEAFF	1
HD2	1501-YF45	TCM	CAVGAQGGFGNVLHC-CASSSTTDGYTF	1
HD2	1501-YF45	TCM	CIVRPLSGNTPLVF-CASSWTGALGEQYF	1
HD2	1501-YF45	TCM	CIVRVASAGNNRKLIW-CASTLSGGVYNEQFF	1
HD2	1501-YF45	TCM	CALVSNSGYALNF-CATSDFPVVGVNYGYTF	1
HD2	1501-YF45	TCM	CIVRVPVVNNNDMRF-CSAISGSVYNEQFF	1
HD2	1501-YF45	TCM	CAFISDGQKLLF-CSAKFTTGRKETQYF	1
HD2	1501-YF45	TCM	CATDAYTDKLIF-CSAKMTSGASYEQYF	1
HD2	1501-YF45	TCM	CLVETGNTGKLIF-CSAQMRTGGSGNTIYF	1
HD2	1501-YF45	TCM	CIVPPGGGADGLTF-CSARALTTVAEAFF	1
HD2	1501-YF45	TCM	CALTFSASKIIF-CSARLLVNGEQFF	1
HD2	1501-YF45	TCM	CIVRLPISGNTPLVF-CSARPPTLGQTNTEAFF	1
HD2	1501-YF45	TCM	CAGHPRGYALNF-CSARRGEASYEQYF	1
HD2	1501-YF45	TCM	CAAP*TAQGGKLIF-CSARRTSILNEQYF;CALDSRDAGKSTF-CSARRTSILNEQYF	1
HD2	1501-YF45	TCM	CAGSSYGQNFVF-CSARSRTGWGTEAFF	1
HD2	1501-YF45	TCM	CVVSEGNAGNMLTF-CSARVLAGGPYVEQYF	1
HD2	1501-YF45	TCM	CAGRPYNFNKFYF-CSARVLSGGPQETQYF	1
HD2	1501-YF45	TCM	CAAHNDMRF-CSARVLTISSYTF	1
HD2	1501-YF45	TCM	CAVDAGGTSYGKLTF-CSARVLVAGELFF	1
HD2	1501-YF45	TCM	CIARNTGFQKLVF-CSARVTAINTGELFF	1
HD2	1501-YF45	TCM	CAENKNQGGKLIF-CSASAGSLGQPQHF	1
HD2	1501-YF45	TCM	CAVVYMEYGNKLVF-CSASFTSGGWTDTQYF	1
HD2	1501-YF45	TCM	CLVGGNTNAGKSTF-CSASLVTGGTGELFF;CLVGGNTNAGKSTF-CSASLVTGGTGELFF	1
HD2	1501-YF45	TCM	WGLPGQ_RALTF-CSASPLTGDEETQYF	1
HD2	1501-YF45	TCM	CAV**ML_NNRKLIW-CSASQGAVGNTIYF	1
HD2	1501-YF45	TCM	CANAGGTSYGKLTF-CSASRGYTGELFF	1
HD2	1501-YF45	TCM	CAENRNQGGKLIF-CSASTGSLGQPQHF	1
HD2	1501-YF45	TCM	CALNYGANNLFF-CSASVLAGGYNEQFF	1
HD2	1501-YF45	TCM	CAENRAAGNKLTF-CSATQMSVGELFF	1
HD2	1501-YF45	TCM	CIVRVLAGANNLFF-CSVAGLYNEQFF	1
HD2	1501-YF45	TCM	CIVRPSAGNTGKLIF-CSVEGTSGRGEQFF	1
HD2	1501-YF45	TCM	CIAIGGATNKLIF-CSVGDRVGTGELFF	1
HD2	1501-YF45	TCM	CIVGSGNTGKLIF-CSVNYRTGMNTEAFF	1
HD2	1501-YF45	TCM	CILRPNYGGSQGNLIF-CASSKTSGLQSYNEQFF;CILRPNYGGSQGNLIF-CASSKTSGLQSYNEQFF	2
HD2	1501-YF45	TCM	CASSTGGLTTEAFF	2
HD2	1501-YF45	TCM	CASSTGGLTTEAFF	2
HD2	1501-YF45	TCM	CAERTSGGYQKVTF-CSAGVRVEGEQFF	2
HD2	1501-YF45	TCM	CSARAITARYEQYF	2
HD2	1501-YF45	TCM	CSARAITARYEQYF	2
HD2	1501-YF45	TCM	CALNYRKTF-CSARVLTGNNQPQHF	2
HD2	1501-YF45	TCM	CAENRNAGNMLTF-CSASQGAVGNTIYF	2

HD2	1501-YF45	TCM	CAENRNAGNMLTF-CSASQGAVGNTIYF	2
HD2	1501-YF45	TCM	CAENKAAGNKLTF-CSASSGTVSGNTIYF	2
HD2	1501-YF45	TCM	CAENKAAGNKLTF-CSASSGTVSGNTIYF	2
HD2	1501-YF45	TCM	CIVRVQTGANNLFF-CASSLAVTVRSSNYGYTF	3
HD2	1501-YF45	TCM	CIVRPSAGNTGKLIF-CASSLGFSMTDRGYTF	3
HD2	1501-YF45	TCM	CAVRDRGGFGNVLHC-CSANPRTGYNQPQHF	3
HD2	1501-YF45	TCM	CAVRDRGGFGNVLHC-CSANPRTGYNQPQHF	3
HD2	1501-YF45	TCM	CAVRDRGGFGNVLHC-CSANPRTGYNQPQHF	3
HD2	1501-YF45	TCM	CSARLSGSIGEQFF	3
HD2	1501-YF45	TCM	CIVRVRAGNTPLVF-CASSLGSLAGSYNEQFF	4
HD2	1501-YF45	TCM	CIVRVAAGQSGYALNF-CASSEGHIPMNTEAFF	5
HD2	1501-YF45	TCM	CIVRSAGNMLTF-CASSSGASTPGYEQYF;CIVRSAGNMLTF-CASSSGASTPGYEQYF	5
HD2	1501-YF45	TCM	CIVRSAGNMLTF-CASSSGASTPGYEQYF	5
HD2	1501-YF45	TCM	CASSSTTDGYTF	5
HD2	1501-YF45	TCM	CASSSTTDGYTF	5
HD2	1501-YF45	TCM	CASSSTTDGYTF	5
HD2	1501-YF45	TCM	CALGGFKTIF-CASSQQQGLNTEAFF	7
HD2	1501-YF45	TCM	CAVQAWDKIIF-CASSVGMGSTDTQYF	7
HD2	1501-YF45	TCM	CIVRVQTGANNLFF-CASTSSGGIYNEQFF	8
HD2	1501-YF45	TCM	CIVRVQTGANNLFF-CASTSSGGIYNEQFF	8
HD2	1501-YF45	TCM	CIVRVQTGANNLFF-CASTSSGGIYNEQFF	8
HD2	1501-YF45	TCM	CIVRVQTGANNLFF-CASTSSGGIYNEQFF	8
HD2	1501-YF45	TCM	CVVSFLW_GYNKLIF-CASIKIGVLGYGYTF	9
HD2	1501-YF45	TCM	CVVSFLW_GYNKLIF-CASIKIGVLGYGYTF	9
HD2	1501-YF45	TCM	CIVRVAAGNTGKLIF-CASQTGASVTYEQYF	9
HD2	1501-YF45	TCM	CIVRVAAGNTGKLIF-CASQTGASVTYEQYF	9
HD2	1501-YF45	TCM	CIVRVAAGNTGKLIF-CASQTGASVTYEQYF	9
HD2	1501-YF45	TCM	CAGRTNTGNQFYF-CASSSMGLAGGLTGELFF;CALVNRDNARLMF-CASSSMGLAGGLTGELFF	12
HD2	1501-YF45	TCM	CAGRTNTGNQFYF-CASSSMGLAGGLTGELFF	12
HD2	1501-YF45	TCM	CAGRTNTGNQFYF-CASSSMGLAGGLTGELFF	12
HD2	1501-YF45	TCM	CAGRTNTGNQFYF-CASSSMGLAGGLTGELFF	12
HD2	1501-YF45	TCM	CAGRTNTGNQFYF-CASSSMGLAGGLTGELFF	12
HD2	1501-YF45	TCM	CAGRTNTGNQFYF-CASSSMGLAGGLTGELFF	12
HD2	1501-YF45	TCM	CIALPAGGTSYGKLTF-CASSPTGLGDYGYTF	15
HD2	1501-YF45	TCM	CIALPAGGTSYGKLTF-CASSPTGLGDYGYTF	15
HD2	1501-YF45	TCM	CIALPAGGTSYGKLTF-CASSPTGLGDYGYTF	15
HD2	1501-YF45	TCM	CIALPAGGTSYGKLTF-CASSPTGLGDYGYTF	15
HD2	1501-YF45	TCM	CIALPAGGTSYGKLTF-CASSPTGLGDYGYTF	15
HD2	1501-YF45	TCM	CIALPAGGTSYGKLTF-CASSPTGLGDYGYTF	15
HD2	1501-YF45	TEM	CAVGSSGNTGKLIF-CASHPGTSIAGELFF	1
HD2	1501-YF45	TEM	CAYRSAGTGNQFYF-CASKMDSWTNTEAFF	1
HD2	1501-YF45	TEM	CIVRVVAGNEKLTF-CASSKGSTSGSYNEQFF	1
HD2	1501-YF45	TEM	CAPPDQAGTALIF-CASSLAGVGPGGYEQFF	1
HD2	1501-YF45	TEM	CAGSAINSGGSNYKLTF-CASSLEPVRDTEAFF	1
HD2	1501-YF45	TEM	CIGKHTGTASKLTF-CASSLGGTVNTEAFF	1
HD2	1501-YF45	TEM	CIVTPLSGYALNF-CASSLGPGLAGETQYF	1
HD2	1501-YF45	TEM	CIGWSSGDKLTF-CASSLGTSYRAPYEQYF	1
HD2	1501-YF45	TEM	CAGSGGSYIPTF-CASSLQM*A_RTTDTQYF	1
HD2	1501-YF45	TEM	CADGSGNTGKLIF-CASSLTQGRNSPLHF	1
HD2	1501-YF45	TEM	CIVRVGAGSARQLTF-CASSQERLTYTGELFF	1
HD2	1501-YF45	TEM	CAMREGPYNFNKFYF-CASSQSRQSAYEQYF	1
HD2	1501-YF45	TEM	CAVGRQGGGADGLTF-CASSSGGLNTEAFF	1
HD2	1501-YF45	TEM	CIVRPSSGNTGKLIF-CASSSGGSVSNEQFF	1
HD2	1501-YF45	TEM	CAVGAQGGGADGLTF-CASSTGGLTTEAFF	1
HD2	1501-YF45	TEM	CAMSPTGGTSYGKLTF-CASSVAGSVNTEAFF	1
HD2	1501-YF45	TEM	CAVRGTSYGKLTF-CASSYEAG_ANNEQFF	1
HD2	1501-YF45	TEM	CILKAPISGNTPLVF-CSAIQGVVWDTQYF	1
HD2	1501-YF45	TEM	CAAPSGNTGKLIF-CSAPARTGTTEQYF	1
HD2	1501-YF45	TEM	CAENRAAGNKLTF-CSAPVGSLGQPQHF	1
HD2	1501-YF45	TEM	CIVRVEGGNNRKLIW-CSARAPAFAYNEQFF	1
HD2	1501-YF45	TEM	CAMTGSARQLTF-CSARAVTGDNSPLHF;CAMTGSARQLTF-CSARAVTGDNSPLHF	1
HD2	1501-YF45	TEM	CIVRVAGRSINTDKLIF-CSARDDGEQFF	1
HD2	1501-YF45	TEM	CIVRGPVGGNKLTF-CSARDLGQSYGYTF	1

HD2	1501-YF45	TEM	CAMSVTNPFHNAGNMLTF-CSARPRTGGEQYF	1
HD2	1501-YF45	TEM	CALSVIQGAQKLVF-CSARTLAGGPGETQYF	1
HD2	1501-YF45	TEM	CAVSDRNGNKLVF-CSARVITGGGEQYF	1
HD2	1501-YF45	TEM	CAGEPYNTDKLIF-CSARVLTYNSPLHF	1
HD2	1501-YF45	TEM	CAVVREETSGSRLTF-CSARVVSGSGIQYF	1
HD2	1501-YF45	TEM	CSARVYVGSEQFF	1
HD2	1501-YF45	TEM	CAASRPDSWGKLQF-CSASARVGGELFF	1
HD2	1501-YF45	TEM	CAPSETDKLIF-CSASAVVGNIQYF	1
HD2	1501-YF45	TEM	CAVKNAGGTSYGKLTF-CSASPLSGGANYEQYF;CAVKNAGGTSYGKLTF-CSASPLSGGANYEQYF	1
HD2	1501-YF45	TEM	CAENMAAGNKLTF-CSASQGTASGNTIYF	1
HD2	1501-YF45	TEM	CAGRFQGAQKLVF-CSASVRTGDNQPQHF	1
HD2	1501-YF45	TEM	CALSEAYGSGNTGKLIF-CSATIRTGAGVEQYF	1
HD2	1501-YF45	TEM	CIVPGTYKYIF-CSATLRTGANTEAFF	1
HD2	1501-YF45	TEM	CAFNGLGDSWGKLQF-CSAYQRVAGELFF	1
HD2	1501-YF45	TEM	CALSGYMGGSEKLVF-CSGKAVSGSNQPQHF	1
HD2	1501-YF45	TEM	CAVRVYSSASKIIF-CSVEDRAGGTEAFF	1
HD2	1501-YF45	TEM	CIVRVPSPSAGNMLTF-CSVGNRVGSRELFF	1
HD2	1501-YF45	TEM	CLVGAGNTGKLIF-CSVSDRVGSDTIYF	1
HD2	1501-YF45	TEM	CIVKGTGTASKLTF-CASSLAGTVNTEAFF	2
HD2	1501-YF45	TEM	CIVSALSGNTGKLIF-CASSLGFVAGKELFF	2
HD2	1501-YF45	TEM	CIVSALSGNTGKLIF-CASSLGFVAGKELFF	2
HD2	1501-YF45	TEM	CIVSRAGSYQLTF-CASSLTAGRGAGGANVLTF;CAFIMYSGGGADGLTF-CASSLTAGRGAGGANVLTF	2
HD2	1501-YF45	TEM	CIVSRAGSYQLTF-CASSLTAGRGAGGANVLTF	2
HD2	1501-YF45	TEM	CAVQQE_TPLVF-CASSPGGLSTEAFF	2
HD2	1501-YF45	TEM	CAVQQE_TPLVF-CASSPGGLSTEAFF	2
HD2	1501-YF45	TEM	CATVDNQGGKLIF-CASSPSRPGGPLSYEQYF	2
HD2	1501-YF45	TEM	CILKTSGSRLTF-CASSTGSTTPNNEQFF	2
HD2	1501-YF45	TEM	CAVSHRSGYSTLTF-CASSTLLAGAPPTDTQYF	2
HD2	1501-YF45	TEM	CIVKNTGTASKLTF-CASSVAGTVNTEAFF	2
HD2	1501-YF45	TEM	CIVKNTGTASKLTF-CASSVAGTVNTEAFF	2
HD2	1501-YF45	TEM	CAGPGDSSYKLIF-CASSYSTGGAAKNIQYF	2
HD2	1501-YF45	TEM	CAGPGDSSYKLIF-CASSYSTGGAAKNIQYF	2
HD2	1501-YF45	TEM	CAERTSGGYQKVTF-CSAGVRVEGEQFF;CAERTSGGYQKVTF-CSAGVRVEGEQFF	2
HD2	1501-YF45	TEM	CAYRSRYPYNTDKLIF-CSARAVVGGYEQYF;CAGGLTQGGSEKLVF-CSARAVVGGYEQYF	2
HD2	1501-YF45	TEM	CAVRDGSAYNTDKLIF-CSARISNLNQETQYF	2
HD2	1501-YF45	TEM	CAVLIGGATNKLIF-CSARTRVGGEQYF;CAFPPRDDKIIF-CSARTRVGGEQYF	2
HD2	1501-YF45	TEM	CAVLIGGATNKLIF-CSARTRVGGEQYF	2
HD2	1501-YF45	TEM	CALNYRKTF-CSARVLTGNNQPQHF;CAENGSGNTGKLIF-CSARVLTGNNQPQHF	2
HD2	1501-YF45	TEM	CAVLLRL_ATNKLIF-CSASARVGGELFF	2
HD2	1501-YF45	TEM	CIVRGNQFYF-CSASPRVEGEQYF	2
HD2	1501-YF45	TEM	CATGYLMGGATNKLIF-CSASYMTGVGTQYF	2
HD2	1501-YF45	TEM	CATGYLMGGATNKLIF-CSASYMTGVGTQYF	2
HD2	1501-YF45	TEM	CASIKIGVLGYGYTF	3
HD2	1501-YF45	TEM	CASIKIGVLGYGYTF	3
HD2	1501-YF45	TEM	CIVRVQTGANNLFF-CASSLAVTVRSSNYGYTF	3
HD2	1501-YF45	TEM	CIVRPSAGNTGKLIF-CASSLGFSMTDRGYTF;CAENNGQLGDKIIF-CASSLGFSMTDRGYTF	3
HD2	1501-YF45	TEM	CIVRPSAGNTGKLIF-CASSLGFSMTDRGYTF	3
HD2	1501-YF45	TEM	CAMREGPYNFNKFYF-CASSLSRQGAREQFF	3
HD2	1501-YF45	TEM	CAMREGPYNFNKFYF-CASSLSRQGAREQFF	3
HD2	1501-YF45	TEM	CVVSVGKFSDGQKLLF-CASSQRQGINTEAFF;CIVRSAGNMLTF-CASSQRQGINTEAFF	3
HD2	1501-YF45	TEM	CVVSVGKFSDGQKLLF-CASSQRQGINTEAFF	3
HD2	1501-YF45	TEM	CAASHGFQKLVF-CSAHQGNGYTF;CIALPAGGTSYGKLTF-CSAHQGNGYTF	3
HD2	1501-YF45	TEM	CAASHGFQKLVF-CSAHQGNGYTF	3
HD2	1501-YF45	TEM	CSARLSGSIGEQFF	3
HD2	1501-YF45	TEM	CIVRVRAGNTPLVF-CASSLGSLAGSYNEQFF	4
HD2	1501-YF45	TEM	CIVRVRAGNTPLVF-CASSLGSLAGSYNEQFF	4
HD2	1501-YF45	TEM	CIVRVAAGQSGYALNF-CASSEGHIPMNTEAFF	5

HD2	1501-YF45	TEM	CIVRVAAGQSGYALNF-CASSEGHIPMNTEAFF	5
HD2	1501-YF45	TEM	CIVRVAAGQSGYALNF-CASSEGHIPMNTEAFF	5
HD2	1501-YF45	TEM	CIVRVAAGQSGYALNF-CASSEGHIPMNTEAFF	5
HD2	1501-YF45	TEM	CIVRSAGNMLTF-CASSSGASTPGYEQYF	5
HD2	1501-YF45	TEM	CASSSTTDGYTF	5
HD2	1501-YF45	TEM	CASSSTTDGYTF	5
HD2	1501-YF45	TEM	CALGGFKTIF-CASSQQQGLNTEAFF;CALGGFKTIF-CASSQQQGLNTEAFF	7
HD2	1501-YF45	TEM	CALGGFKTIF-CASSQQQGLNTEAFF;CALGGFKTIF-CASSQQQGLNTEAFF	7
HD2	1501-YF45	TEM	CALGGFKTIF-CASSQQQGLNTEAFF;CALGGFKTIF-CASSQQQGLNTEAFF	7
HD2	1501-YF45	TEM	CALGGFKTIF-CASSQQQGLNTEAFF	7
HD2	1501-YF45	TEM	CALGGFKTIF-CASSQQQGLNTEAFF	7
HD2	1501-YF45	TEM	CAVQAWDKIIF-CASSVGMGSTDTQYF	7
HD2	1501-YF45	TEM	CAVQAWDKIIF-CASSVGMGSTDTQYF	7
HD2	1501-YF45	TEM	CAVQAWDKIIF-CASSVGMGSTDTQYF	7
HD2	1501-YF45	TEM	CAVQAWDKIIF-CASSVGMGSTDTQYF	7
HD2	1501-YF45	TEM	CAVQAWDKIIF-CASSVGMGSTDTQYF	7
HD2	1501-YF45	TEM	CIVRVQTGANNLFF-CASTSSGGIYNEQFF	8
HD2	1501-YF45	TEM	CIVRVQTGANNLFF-CASTSSGGIYNEQFF	8
HD2	1501-YF45	TEM	CIVRVQTGANNLFF-CASTSSGGIYNEQFF	8
HD2	1501-YF45	TEM	CIVRVQTGANNLFF-CASTSSGGIYNEQFF	8
HD2	1501-YF45	TEM	CVVSFLW_GYNKLIF-CASIKIGVLGYGYTF	9
HD2	1501-YF45	TEM	CVVSFLW_GYNKLIF-CASIKIGVLGYGYTF	9
HD2	1501-YF45	TEM	CVVSFLW_GYNKLIF-CASIKIGVLGYGYTF	9
HD2	1501-YF45	TEM	CIVRVAAGNTGKLIF-CASQTGASVTYEQYF	9
HD2	1501-YF45	TEM	CAGRTNTGNQFYF-CASSSMGLAGGLTGELFF;CALVNRDNARLMF-CASSSMGLAGGLTGELFF	12
HD2	1501-YF45	TEM	CAGRTNTGNQFYF-CASSSMGLAGGLTGELFF;CALVNRDNARLMF-CASSSMGLAGGLTGELFF	12
HD2	1501-YF45	TEM	CAGRTNTGNQFYF-CASSSMGLAGGLTGELFF;CAGRTNTGNQFYF-CASSSMGLAGGLTGELFF	12
HD2	1501-YF45	TEM	CAGRTNTGNQFYF-CASSSMGLAGGLTGELFF;CALVNRDNARLMF-CASSSMGLAGGLTGELFF	12
HD2	1501-YF45	TEM	CAGRTNTGNQFYF-CASSSMGLAGGLTGELFF	12
HD2	1501-YF45	TEM	CIALPAGGTSYGKLTF-CASSPTGLGDYGYTF	15
HD2	1501-YF45	TEM	CIALPAGGTSYGKLTF-CASSPTGLGDYGYTF	15
HD2	1501-YF45	TEM	CIALPAGGTSYGKLTF-CASSPTGLGDYGYTF	15
HD2	1501-YF45	TEMRA	CAASAISNSGGSNYKLTF-CASSLSALSGNTIYF;CAMREGPYNFNKFYF-CASSLSALSGNTIYF	1
HD2	1501-YF45	TEMRA	CAFRNTGNQFYF-CASSPGTSVADTQYF;CAAPIKAAGNKLTF-CASSPGTSVADTQYF	1
HD2	1501-YF45	TEMRA	CIVLRPPGNTPLVF-CSARESLAGAREQYF	1
HD2	1501-YF45	TEMRA	CILKAPISGNTPLVF-CSARMDSLNTGELFF	1
HD2	1501-YF45	TEMRA	CAPDR_NQFYF-CSARSRTGWGTEAFF	1
HD2	1501-YF45	TEMRA	CAVYNDMRF-CSARVLTNEKLFF	1
HD2	1501-YF45	TEMRA	CILKTSGSRLTF-CASSTGSTTPNNEQFF	2
HD2	1501-YF45	TEMRA	CAVSHRSGYSTLTF-CASSTLLAGAPPTDTQYF;CATDAIQ_DYKLSF-CASSTLLAGAPPTDTQYF	2
HD2	1501-YF45	TEMRA	CAYRSRYPYNTDKLIF-CSARAVVGGYEQYF;CAGGLTQGGSEKLVF-CSARAVVGGYEQYF	2
HD2	1501-YF45	TEMRA	CAVRDGSAYNTDKLIF-CSARISNLNQETQYF	2
HD2	1501-YF45	TEMRA	CASIKIGVLGYGYTF	3
HD2	1501-YF45	TEMRA	CSARLSGSIGEQFF	3
HD2	1501-YF45	TEMRA	CIVRSAGNMLTF-CASSSGASTPGYEQYF	5
HD2	1501-YF45	TEMRA	CAVQAWDKIIF-CASSVGMGSTDTQYF	7
HD2	1501-YF45	TEMRA	CVVSFLW_GYNKLIF-CASIKIGVLGYGYTF	9
HD2	1501-YF45	TEMRA	CIALPAGGTSYGKLTF-CASSPTGLGDYGYTF	15
HD2	1501-YF45	TEMRA	CIALPAGGTSYGKLTF-CASSPTGLGDYGYTF	15
HD2	1501-YF45	TEMRA	CIALPAGGTSYGKLTF-CASSPTGLGDYGYTF	15
HD2	1501-YF45	TMN	CALSPFSGGYNKLIF-CASSEQSLNNYNEQFF	1
HD2	1501-YF45	TMN	CAAKGLK_SGYALNF-CASSPRQGLNTEAFF	1
HD2	1501-YF45	TMN	CAGEVLV_YNKLIF-CASSSTTDGYTF	1
HD2	1501-YF45	TMN	CIVKGTGTASKLTF-CASSLAGTVNTEAFF	2
HD2	1501-YF45	TMN	CIVRVQTGANNLFF-CASSLAVTVRSSNYGYTF	3
HD2	1501-YF45	TMN	CAASHGFQKLVF-CSAHQGNGYTF	3
HD2	1501-YF45	TMN	CALGGFKTIF-CASSQQQGLNTEAFF	7
HD2	1501-YF45	TMN	CVVSFLW_GYNKLIF-CASIKIGVLGYGYTF	9

HD2	1501-YF45	TMN	CVVSFLW_GYNKLIF-CASIKIGVLGYGYTF	9
HD2	1501-YF45	TSCM	CIVRVANSGNTPLVF-CSARVPQQGLAHNEQFF	1
HD2	1501-YF45	TSCM	CAGTLTF-CSVSDRVGWDTEAFF	1
HD2	1501-YF45	TSCM	CILRPNYGGSQGNLIF-CASSKTSGLQSYNEQFF	2
HD2	1501-YF45	TSCM	CIVRVRAGNTPLVF-CASSLGSLAGSYNEQFF	4
HD2	1501-YF45	TSCM	CIVRSAGNMLTF-CASSSGASTPGYEQYF	5
HD2	1501-YF45	TSCM	CVVSFLW_GYNKLIF-CASIKIGVLGYGYTF	9
HD2	1501-YF45	TSCM	CIVRVAAGNTGKLIF-CASQTGASVTYEQYF	9
HD2	1501-YF45	TSCM	CIVRVAAGNTGKLIF-CASQTGASVTYEQYF	9
HD2	1501-YF45	TSCM	CAGRTNTGNQFYF-CASSSMGLAGGLTGELFF	12
HD3	0407-YF54	Naive	CAVNNRDDKIIF-CASSFLAGANEQFF	1
HD3	0407-YF54	Naive	CASSLLGQTEAFF	1
HD3	0407-YF54	Naive	CAVEGQGGGYNKLIF-CSARHLQGNRNTEAFF	2
HD3	0407-YF54	Naive	CAVNGYGNKLVF-CSARDWQGQRNTEAFF	3
HD3	0407-YF54	Naive	CAVNSNFNKFYF-CSARDWQGARNTEAFF	4
HD3	0407-YF54	Naive	CAVNSNFNKFYF-CSARDWQGARNTEAFF	4
HD3	0407-YF54	Naive	CAVNGGDDKIIF-CSAREIQGARNTEAFF	7
HD3	0407-YF54	Naive	CAVNNRDDKIIF-CSARVVQGRRNTEAFF	14
HD3	0407-YF54	Naive	CAVNNRDDKIIF-CSARVVQGRRNTEAFF	14
HD3	0407-YF54	TCM	CAGEKL_GNKLTF-CASSPGTGDGYTF	1
HD3	0407-YF54	TCM	CASSPQGQRPYEQYV	1
HD3	0407-YF54	TCM	CAVNDRDDKIIF-CASSQIGTSGSFSYNEQFF	1
HD3	0407-YF54	TCM	CATSREVSRGQYF	1
HD3	0407-YF54	TCM	CSARDEGAKNIQYF	1
HD3	0407-YF54	TCM	CSARVADRGTTRNTEAFF	1
HD3	0407-YF54	TCM	CALIGDDMRF-CASSPTSGRGYEQYF	2
HD3	0407-YF54	TCM	CAVNRGDDKIIF-CSARVADRGTTRNTEAFF	2
HD3	0407-YF54	TCM	CAVNGYGNKLVF-CSARDWQGQRNTEAFF	3
HD3	0407-YF54	TCM	CAVNDRDDKIIF-CSARGLQRRNTEAFF	3
HD3	0407-YF54	TCM	CAVNGGDDKIIF-CSAREIQGARNTEAFF	7
HD3	0407-YF54	TCM	CAVNGGDDKIIF-CSAREIQGARNTEAFF	7
HD3	0407-YF54	TCM	CAVNGGDDKIIF-CSAREIQGARNTEAFF	7
HD3	0407-YF54	TCM	CAVNGGDDKIIF-CSAREIQGARNTEAFF	7
HD3	0407-YF54	TCM	CAVNNRDDKIIF-CSARVVQGRRNTEAFF	14
HD3	0407-YF54	TEM	CAGRNGGSQGNLIF-CASSFGTGVDEAFF	1
HD3	0407-YF54	TEM	CAVREDDKIIF-CASSLAGGFYEQYF	1
HD3	0407-YF54	TEM	CAVSGRTGAGSYQLTF-CASSPQGPSYEQYF	1
HD3	0407-YF54	TEM	CAFMKPFPGGTSYGKLTF-CASSPSLGDTQYF	1
HD3	0407-YF54	TEM	CASSQSIDRNNYGYTF	1
HD3	0407-YF54	TEM	CSARDLQGARNTEAFF	1
HD3	0407-YF54	TEM	CAVTLNNNDMRF-CSARDRQGRNTEAFF	1
HD3	0407-YF54	TEM	CAVNMGDDKIIF-CSARDVQGRRNTEAFF	1
HD3	0407-YF54	TEM	CSARGTGGAKSYTF	1
HD3	0407-YF54	TEM	CAVNNGGNKLVF-CASSNGA_NTGELFF	2
HD3	0407-YF54	TEM	CALIGDDMRF-CASSPTSGRGYEQYF	2
HD3	0407-YF54	TEM	CAVNRGDDKIIF-CSARVADRGTTRNTEAFF	2
HD3	0407-YF54	TEM	CSARVVQGRRNTEAFF	2
HD3	0407-YF54	TEM	CAVNGYGNKLVF-CSARDWQGQRNTEAFF	3
HD3	0407-YF54	TEM	CAVNDRDDKIIF-CSARGLQRRNTEAFF	3
HD3	0407-YF54	TEM	CAVNDRDDKIIF-CSARGLQRRNTEAFF	3
HD3	0407-YF54	TEM	CAVNSNFNKFYF-CSARDWQGARNTEAFF	4
HD3	0407-YF54	TEM	CAVNSNFNKFYF-CSARDWQGARNTEAFF	4
HD3	0407-YF54	TEM	CAVNGGDDKIIF-CSAREIQGARNTEAFF	7
HD3	0407-YF54	TEM	CAVNGGDDKIIF-CSAREIQGARNTEAFF	7
HD3	0407-YF54	TEM	CAVNNRDDKIIF-CSARVVQGRRNTEAFF	14
HD3	0407-YF54	TEM	CAVNNRDDKIIF-CSARVVQGRRNTEAFF	14
HD3	0407-YF54	TEM	CAVNNRDDKIIF-CSARVVQGRRNTEAFF	14
HD3	0407-YF54	TEM	CAVNNRDDKIIF-CSARVVQGRRNTEAFF	14
HD3	0407-YF54	TEM	CAVNNRDDKIIF-CSARVVQGRRNTEAFF	14
HD3	0407-YF54	TEM	CAVNNRDDKIIF-CSARVVQGRRNTEAFF	14
HD3	0407-YF54	TEM	CAVNNRDDKIIF-CSARVVQGRRNTEAFF	14
HD3	0407-YF54	TEM	CAVNNRDDKIIF-CSARVVQGRRNTEAFF	14
HD3	0407-YF54	TEM	CAVNNRDDKIIF-CSARVVQGRRNTEAFF	14
HD3	0407-YF54	TEM	CAVNNRDDKIIF-CSARVVQGRRNTEAFF	14
HD3	0407-YF54	TEMRA	CASSIGTARVEKLFF	1
HD3	0407-YF54	TEMRA	CAVPLRSNDYKLSF-CSARDYQGDRNTEAFF	1

HD3	0407-YF54	TEMRA	CAVNNGGNKLVF-CASSNGA_NTGELFF	2
HD3	0407-YF54	TEMRA	CAVEGQGGGYNKLIF-CSARHLQGNRNTEAFF	2
HD3	0407-YF54	TEMRA	CSARVVQGRRNTEAFF	2
HD3	0407-YF54	TEMRA	CAVNNRDDKIIF-CSARVVQGRRNTEAFF	14
HD3	1501-YF45	Naive	CALIGDDMRF-CAWSLRQAAAPLHF;CALSEGANTGGFKTIF-CAWSLRQAAAPLHF	1
HD3	1501-YF45	Naive	CIVRNNAGNMLTF-CSARATSGGASEQYF	1
HD3	1501-YF45	Naive	CAVHSGNTPLVF-CSARVGSLADTQYF	1
HD3	1501-YF45	Naive	CAMSHTGFQKLVF-CASSVGSVSGGTGELFF	2
HD3	1501-YF45	Naive	CSAKMRVGGELFF	2
HD3	1501-YF45	Naive	CIVRVGNYGQNFVF-CASSSGLAIEQYF	3
HD3	1501-YF45	Naive	CAASGAL_GYNKLIF-CSARATSGGASEQYF	3
HD3	1501-YF45	Naive	CAGAGGTSYGKLTF-CASSMTVQGAIGANVLTF	5
HD3	1501-YF45	Naive	CIVRNNAGNMLTF-CASSPQGPLINEQFF	14
HD3	1501-YF45	Naive	CAGQGYNQGGKLIF-CSASYLTGSYNEQFF	49
HD3	1501-YF45	Naive	CAGQGYNQGGKLIF-CSASYLTGSYNEQFF	49
HD3	1501-YF45	Naive	CAGQGYNQGGKLIF-CSASYLTGSYNEQFF	49
HD3	1501-YF45	Naive	CAGQGYNQGGKLIF-CSASYLTGSYNEQFF	49
HD3	1501-YF45	TCM	CIVRVAADYKLSF-CASSFGTVVDTEAFF	1
HD3	1501-YF45	TCM	CIVRVGAQGAQKLVF-CASSFPRGQNINQPQHF	1
HD3	1501-YF45	TCM	CVVIPNW_ANNLFF-CASSLEAGLSTDTQYF	1
HD3	1501-YF45	TCM	CALSDRDTGNQFYF-CASSLGASGGAAGEQFF	1
HD3	1501-YF45	TCM	CAAGT**_AGNMLTF-CASSLGDQVSNQPQHF	1
HD3	1501-YF45	TCM	CIVRVVTDYKLSF-CASSLQGPLSYEQYF	1
HD3	1501-YF45	TCM	CASSQGGLNTEAFF	1
HD3	1501-YF45	TCM	CAENTNTGGFKTIF-CASSRDRGPYEQYF	1
HD3	1501-YF45	TCM	CASSRNGGPFSEQYF	1
HD3	1501-YF45	TCM	CASSSSGGIYNEQFF	1
HD3	1501-YF45	TCM	CAENNNNARLMF-CASSTQGLITEAFF	1
HD3	1501-YF45	TCM	CIAKATGTASKLTF-CASSYPGTANTEAFF	1
HD3	1501-YF45	TCM	CAAGGRGGNTGKLIF-CSALVRTGDQQPQHF	1
HD3	1501-YF45	TCM	CAAIRGTYKYIF-CSANVRVEGEQYF	1
HD3	1501-YF45	TCM	CATDARTTDSWGKLQF-CSARILTSNNSPLHF	1
HD3	1501-YF45	TCM	CAAFAYSGAGSYQLTF-CSARVGSVIGNTIYF	1
HD3	1501-YF45	TCM	CAASGAGGTSYGKLTF-CSARVIAGAYEQYF	1
HD3	1501-YF45	TCM	CAENTPQAGTALIF-CSARVSGFNEQFF	1
HD3	1501-YF45	TCM	CIVRSINNAGNMLTF-CSARVVTGSDQPQHF	1
HD3	1501-YF45	TCM	CALDNAGGTSYGKLTF-CSARVVVGDTQYF	1
HD3	1501-YF45	TCM	CAYRSSDYKLSF-CSASVVTNQPQHF	1
HD3	1501-YF45	TCM	CAENGNAGNMLTF-CSATTGSNSGNTIYF	1
HD3	1501-YF45	TCM	CIIHGSSNTGKLIF-CSVEVRVGGTEAFF	1
HD3	1501-YF45	TCM	CALCTGGGNKLTF-CASSSPGLNTEAFF	2
HD3	1501-YF45	TCM	CALCTGGGNKLTF-CASSSPGLNTEAFF	2
HD3	1501-YF45	TCM	CIVRVQTGANNLFF-CASTKSGGVYNEQFF	2
HD3	1501-YF45	TCM	CSAKMRVGGELFF	2
HD3	1501-YF45	TCM	CSARILTGEDSPLHF	2
HD3	1501-YF45	TCM	CIVRVAAEAAGNKLTF-CASRDLYGYTF	3
HD3	1501-YF45	TCM	CIVRVAAEAAGNKLTF-CASRDLYGYTF	3
HD3	1501-YF45	TCM	CASSLEAGLSTDTQYF	3
HD3	1501-YF45	TCM	CIVRVGNYGQNFVF-CASSSGLAIEQYF	3
HD3	1501-YF45	TCM	CIVKVQTGANNLFF-CASSSSGGIYNEQFF	3
HD3	1501-YF45	TCM	CIVKVQTGANNLFF-CASSSSGGIYNEQFF	3
HD3	1501-YF45	TCM	CAASGAL_GYNKLIF-CSARATSGGASEQYF	3
HD3	1501-YF45	TCM	CAGAGGTSYGKLTF-CASSMTVQGAIGANVLTF	5
HD3	1501-YF45	TCM	CSASYLTGSYNEQFF	6
HD3	1501-YF45	TCM	CSASYLTGSYNEQFF	6
HD3	1501-YF45	TCM	CASSTQGLITEAFF	7
HD3	1501-YF45	TCM	CASSTQGLITEAFF	7
HD3	1501-YF45	TCM	CASSTQGLITEAFF	7
HD3	1501-YF45	TCM	CSARATSGGASEQYF	9
HD3	1501-YF45	TCM	CSARATSGGASEQYF	9
HD3	1501-YF45	TCM	CSARATSGGASEQYF	9
HD3	1501-YF45	TCM	CSARATSGGASEQYF	9
HD3	1501-YF45	TCM	CSARATSGGASEQYF	9
HD3	1501-YF45	TCM	CSVEGTSGRGEQFF	12
HD3	1501-YF45	TCM	CSVEGTSGRGEQFF	12

HD3	1501-YF45	TCM	CIVRNNAGNMLTF-CASSPQGPLINEQFF	14
HD3	1501-YF45	TCM	CIVRNNAGNMLTF-CASSPQGPLINEQFF	14
HD3	1501-YF45	TCM	CIVRNNAGNMLTF-CASSPQGPLINEQFF	14
HD3	1501-YF45	TCM	CIVRNNAGNMLTF-CASSPQGPLINEQFF	14
HD3	1501-YF45	TCM	CIVRNNAGNMLTF-CASSPQGPLINEQFF	14
HD3	1501-YF45	TCM	CIVRNNAGNMLTF-CASSPQGPLINEQFF	14
HD3	1501-YF45	TCM	CIVRNNAGNMLTF-CASSPQGPLINEQFF	14
HD3	1501-YF45	TCM	CIVRNNAGNMLTF-CASSPQGPLINEQFF	14
HD3	1501-YF45	TCM	CIVRNNAGNMLTF-CASSPQGPLINEQFF	14
HD3	1501-YF45	TCM	CAGQGYNQGGKLIF-CSASYLTGSYNEQFF	49
HD3	1501-YF45	TCM	CAGQGYNQGGKLIF-CSASYLTGSYNEQFF	49
HD3	1501-YF45	TCM	CAGQGYNQGGKLIF-CSASYLTGSYNEQFF	49
HD3	1501-YF45	TCM	CAGQGYNQGGKLIF-CSASYLTGSYNEQFF	49
HD3	1501-YF45	TCM	CAGQGYNQGGKLIF-CSASYLTGSYNEQFF	49
HD3	1501-YF45	TCM	CAGQGYNQGGKLIF-CSASYLTGSYNEQFF	49
HD3	1501-YF45	TCM	CAGQGYNQGGKLIF-CSASYLTGSYNEQFF	49
HD3	1501-YF45	TCM	CAGQGYNQGGKLIF-CSASYLTGSYNEQFF	49
HD3	1501-YF45	TCM	CAGQGYNQGGKLIF-CSASYLTGSYNEQFF	49
HD3	1501-YF45	TCM	CAGQGYNQGGKLIF-CSASYLTGSYNEQFF	49
HD3	1501-YF45	TCM	CAGQGYNQGGKLIF-CSASYLTGSYNEQFF	49
HD3	1501-YF45	TCM	CAGQGYNQGGKLIF-CSASYLTGSYNEQFF	49
HD3	1501-YF45	TCM	CAGQGYNQGGKLIF-CSASYLTGSYNEQFF	49
HD3	1501-YF45	TCM	CAGQGYNQGGKLIF-CSASYLTGSYNEQFF	49
HD3	1501-YF45	TCM	CAGQGYNQGGKLIF-CSASYLTGSYNEQFF	49
HD3	1501-YF45	TCM	CAGQGYNQGGKLIF-CSASYLTGSYNEQFF	49
HD3	1501-YF45	TCM	CAGQGYNQGGKLIF-CSASYLTGSYNEQFF	49
HD3	1501-YF45	TCM	CAGQGYNQGGKLIF-CSASYLTGSYNEQFF	49
HD3	1501-YF45	TCM	CAGQGYNQGGKLIF-CSASYLTGSYNEQFF	49
HD3	1501-YF45	TCM	CAGQGYNQGGKLIF-CSASYLTGSYNEQFF	49
HD3	1501-YF45	TCM	CAGQGYNQGGKLIF-CSASYLTGSYNEQFF	49
HD3	1501-YF45	TCM	CAGQGYNQGGKLIF-CSASYLTGSYNEQFF	49
HD3	1501-YF45	TEM	CIVKVQTGANNLFF-CASDLRDR_VRYYGYTF	1
HD3	1501-YF45	TEM	CALSEGPNNAGNMLTF-CASSNLRGSEQYF	1
HD3	1501-YF45	TEM	CIPPLA_DDKIIF-CASSSRQGLNTEAFF	1
HD3	1501-YF45	TEM	CAACSNDYKLSF-CSAPVIVGGEQYF	1
HD3	1501-YF45	TEM	CATVNAGGTSYGKLTF-CSARLLSSYNSPLHF	1
HD3	1501-YF45	TEM	CSARVGSLPGNTIYF	1
HD3	1501-YF45	TEM	CALTYSGYSTLTF-CSARVLVGQETQYF	1
HD3	1501-YF45	TEM	CGADGNTGNQFYF-CSARVRVEGEQYF	1
HD3	1501-YF45	TEM	CARDSSGGSNYKLTF-CSARVRVQGTQYF	1
HD3	1501-YF45	TEM	CALTLNSGNTPLVF-CSVEGTSGRGEQFF	1
HD3	1501-YF45	TEM	CAGQEGIQ_GGADGLTF-CSVRTGQVTGELFF	1
HD3	1501-YF45	TEM	CIVRVYAGNMLTF-CASSLVQLNTEAFF	2
HD3	1501-YF45	TEM	CAMSHTGFQKLVF-CASSVGSVSGGTGELFF	2
HD3	1501-YF45	TEM	CSARILTGEDSPLHF	2
HD3	1501-YF45	TEM	CAGQGYNQGGKLIF-CSVEGTSGRGEQFF	2
HD3	1501-YF45	TEM	CAGQGYNQGGKLIF-CSVEGTSGRGEQFF	2
HD3	1501-YF45	TEM	CIVRVAAEAAGNKLTF-CASRDLYGYTF	3
HD3	1501-YF45	TEM	CAGAGGTSYGKLTF-CASSMTVQGAIGANVLTF	5
HD3	1501-YF45	TEM	CASSTQGLITEAFF	7
HD3	1501-YF45	TEM	CASSTQGLITEAFF	7
HD3	1501-YF45	TEM	CASSTQGLITEAFF	7
HD3	1501-YF45	TEM	CSARATSGGASEQYF	9
HD3	1501-YF45	TEM	CSVEGTSGRGEQFF	12
HD3	1501-YF45	TEM	CSVEGTSGRGEQFF	12
HD3	1501-YF45	TEM	CSVEGTSGRGEQFF	12
HD3	1501-YF45	TEM	CSVEGTSGRGEQFF	12
HD3	1501-YF45	TEM	CSVEGTSGRGEQFF	12
HD3	1501-YF45	TEM	CSVEGTSGRGEQFF	12
HD3	1501-YF45	TEM	CIVRNNAGNMLTF-CASSPQGPLINEQFF	14
HD3	1501-YF45	TEM	CAGQGYNQGGKLIF-CSASYLTGSYNEQFF	49
HD3	1501-YF45	TEM	CAGQGYNQGGKLIF-CSASYLTGSYNEQFF	49
HD3	1501-YF45	TEM	CAGQGYNQGGKLIF-CSASYLTGSYNEQFF	49
HD3	1501-YF45	TEM	CAGQGYNQGGKLIF-CSASYLTGSYNEQFF	49
HD3	1501-YF45	TEM	CAGQGYNQGGKLIF-CSASYLTGSYNEQFF	49

HD3	1501-YF45	TEM	CAGQGYNQGGKLIF-CSASYLTGSYNEQFF	49
HD3	1501-YF45	TEM	CAGQGYNQGGKLIF-CSASYLTGSYNEQFF	49
HD3	1501-YF45	TEM	CAGQGYNQGGKLIF-CSASYLTGSYNEQFF	49
HD3	1501-YF45	TEM	CAGQGYNQGGKLIF-CSASYLTGSYNEQFF	49
HD3	1501-YF45	TEM	CAGQGYNQGGKLIF-CSASYLTGSYNEQFF	49
HD3	1501-YF45	TEMRA	CASSHRGLEQPQHF	1
HD3	1501-YF45	TEMRA	CSARVIVAGELFF	1
HD3	1501-YF45	TEMRA	CSARVQTSGEQYF	1
HD3	1501-YF45	TEMRA	CAASGRAQKLVF-CSASIRTGDNQPQHF	1
HD3	1501-YF45	TEMRA	CIVRVGNYGQNFVF-CASSSGLAIEQYF	3
HD3	1501-YF45	TEMRA	CIVKVQTGANNLFF-CASSSSGGIYNEQFF	3
HD3	1501-YF45	TEMRA	CAASGAL_GYNKLIF-CSARATSGGASEQYF	3
HD3	1501-YF45	TEMRA	CSASYLTGSYNEQFF	6
HD3	1501-YF45	TEMRA	CSASYLTGSYNEQFF	6
HD3	1501-YF45	TEMRA	CSASYLTGSYNEQFF	6
HD3	1501-YF45	TEMRA	CASSTQGLITEAFF	7
HD3	1501-YF45	TEMRA	CSARATSGGASEQYF	9
HD3	1501-YF45	TEMRA	CSVEGTSGRGEQFF	12
HD3	1501-YF45	TEMRA	CIVRNNAGNMLTF-CASSPQGPLINEQFF	14
HD3	1501-YF45	TEMRA	CIVRNNAGNMLTF-CASSPQGPLINEQFF	14
HD3	1501-YF45	TEMRA	CAGQGYNQGGKLIF-CSASYLTGSYNEQFF	49
HD3	1501-YF45	TEMRA	CAGQGYNQGGKLIF-CSASYLTGSYNEQFF	49
HD3	1501-YF45	TEMRA	CAGQGYNQGGKLIF-CSASYLTGSYNEQFF	49
HD3	1501-YF45	TEMRA	CAGQGYNQGGKLIF-CSASYLTGSYNEQFF	49
HD3	1501-YF45	TEMRA	CAGQGYNQGGKLIF-CSASYLTGSYNEQFF	49
HD3	1501-YF45	TEMRA	CAGQGYNQGGKLIF-CSASYLTGSYNEQFF	49
HD3	1501-YF45	TEMRA	CAGQGYNQGGKLIF-CSASYLTGSYNEQFF	49
HD3	1501-YF45	TMN	CAMRGSGGGADGLTF-CASIIGTP_SGNTIYF	1
HD3	1501-YF45	TMN	CIVRPSSGNTPLVF-CASSPGSSTSSYNEQFF	1
HD3	1501-YF45	TMN	KGRDDKIIF-CASTLSGGLYNEQFF	1
HD3	1501-YF45	TMN	CIVRVYAGNMLTF-CASSLVQLNTEAFF	2
HD3	1501-YF45	TMN	CIVRVQTGANNLFF-CASTKSGGVYNEQFF	2
HD3	1501-YF45	TMN	CAENNNNARLMF-CSVRTGQVTGELFF	2
HD3	1501-YF45	TMN	CAGAGGTSYGKLTF-CASSMTVQGAIGANVLTF	5
HD3	1501-YF45	TMN	CSARATSGGASEQYF	9
HD3	1501-YF45	TMN	CSVEGTSGRGEQFF	12
HD3	1501-YF45	TMN	CAGQGYNQGGKLIF-CSASYLTGSYNEQFF	49
HD3	1501-YF45	TMN	CAGQGYNQGGKLIF-CSASYLTGSYNEQFF	49
HD3	1501-YF45	TMN	CAGQGYNQGGKLIF-CSASYLTGSYNEQFF	49
HD3	1501-YF45	TSCM	CVVSAVGGTYKYIF-CASSLALRDRGSIQPQHF	1
HD3	1501-YF45	TSCM	CIVRVVTNAGKSTF-CASSLGFMNTEAFF	1
HD3	1501-YF45	TSCM	CIVREAGGFKTIF-CASSLHGGTVSTEAFF	1
HD3	1501-YF45	TSCM	CIVRVGAGGTSYGKLTF-CASTPTGTVGGTQYF	1
HD3	1501-YF45	TSCM	CALSEAGL_FGNEKLTF-CSAKMRVGGELFF	1
HD3	1501-YF45	TSCM	CAVLPGR_GNKLTF-CSALDRVGGEQYF	1
HD3	1501-YF45	TSCM	CLVGATGSARQLTF-CSARGQGVLGELFF	1
HD3	1501-YF45	TSCM	CAMVTSHGKLTF-CSARLSDGTMRSEAFF	1
HD3	1501-YF45	TSCM	CAVANDMRF-CSARVQTSGPQHF	1
HD3	1501-YF45	TSCM	CIAGGNTDKLIF-CSARVVSNQPQHF	1
HD3	1501-YF45	TSCM	CIVGSSNTGKLIF-CSARYVTGNTGELFF	1
HD3	1501-YF45	TSCM	CSARALSTGEQYF	2
HD3	1501-YF45	TSCM	CSARALSTGEQYF	2
HD3	1501-YF45	TSCM	CAENNNNARLMF-CSVRTGQVTGELFF	2
HD3	1501-YF45	TSCM	CASSLEAGLSTDTQYF	3
HD3	1501-YF45	TSCM	CASSLEAGLSTDTQYF	3
HD3	1501-YF45	TSCM	CAGAGGTSYGKLTF-CASSMTVQGAIGANVLTF	5
HD3	1501-YF45	TSCM	CSASYLTGSYNEQFF	6
HD3	1501-YF45	TSCM	CSARATSGGASEQYF	9
HD3	1501-YF45	TSCM	CIVRNNAGNMLTF-CASSPQGPLINEQFF	14
HD3	1501-YF45	TSCM	CAGQGYNQGGKLIF-CSASYLTGSYNEQFF	49
HD3	1501-YF45	TSCM	CAGQGYNQGGKLIF-CSASYLTGSYNEQFF	49
HD3	1501-YF91	Naive	CALSEGDQGGKLIF-CASRPEDEPQHF	1
HD3	1501-YF91	Naive	CASSLDFNSPLHF	1
HD3	1501-YF91	Naive	CAESQGGYQKVTF-CASSLSAGGRDEQFF	1
HD3	1501-YF91	Naive	CAFMIDNARLMF-CASSPFLGAVDTQYF	1

HD3	1501-YF91	Naive	CAVGNQFYF-CASSQDRSGGSYNSPLHF;CAVVTGNQFYF-CASSQDRSGGSYNSPLHF	1
HD3	1501-YF91	Naive	CALSEGDQGGKLIF-CASSQDVGGEIGNSPLHF;CAQIGFGNVLHC-CASSQDVGGEIGNSPLHF	1
HD3	1501-YF91	Naive	CASSQDVGGEIGNSPLHF	1
HD3	1501-YF91	Naive	CASSQEGGGYEQYF	1
HD3	1501-YF91	Naive	CAQIGFGNVLHC-CASSQGTGGVVVEKLFF	1
HD3	1501-YF91	Naive	CALSDMDSSYKLIF-CASSSDRGGSNQPQHF	1
HD3	1501-YF91	Naive	CVTGQGGANNLFF-CATSVRETQYF	1
HD3	1501-YF91	Naive	CAFVPFGGAQKLVF-CAVFTVEAGRDEAFF	2
HD3	1501-YF91	Naive	CAVGAR_TYKYIF-CASSQDLGFENSPLHF	3
HD3	1501-YF91	Naive	CIVRVERDDKIIF-CSAWDRANYGYTF	4
HD3	1501-YF91	Naive	CIVRVERDDKIIF-CSAWDRANYGYTF	4
HD3	1501-YF91	Naive	CALSEGDQGGKLIF-CASASVAGGTYEQYF	6
HD3	1501-YF91	Naive	CALSEGDQGGKLIF-CASASVAGGTYEQYF	6
HD3	1501-YF91	Naive	CAAPLSGSARQLTF-CSARGRIANYGYTF;CAASFNAGNNRKLIW-CSARGRIANYGYTF	7
HD3	1501-YF91	Naive	CAAPLSGSARQLTF-CSARGRIANYGYTF	7
HD3	1501-YF91	Naive	CAAPLSGSARQLTF-CSARGRIANYGYTF	7
HD3	1501-YF91	Naive	CAAPLSGSARQLTF-CSARGRIANYGYTF	7
HD3	1501-YF91	TCM	CASKHRPDSYEQYF	1
HD3	1501-YF91	TCM	CVVSEVQGYGGSQGNLIF-CASSEDSWRGGSTDTQYF	1
HD3	1501-YF91	TCM	CIVRVRGGNNDMRF-CASSFQTGGIVTDTQYF	1
HD3	1501-YF91	TCM	CAVPRVEWYGGATNKLIF-CASSGSIELSGYTF	1
HD3	1501-YF91	TCM	CALSEGDQGGKLIF-CASSQDLAGGSYNSPLHF	1
HD3	1501-YF91	TCM	CASSQDLGFENSPLHF	1
HD3	1501-YF91	TCM	CAGTDRGSTLGRLYF-CASSSSTYEQYF;CAGAPGGR_GADGLTF-CASSSSTYEQYF	1
HD3	1501-YF91	TCM	CLVGDIDNAGNMLTF-CASSVIDNEQFF	1
HD3	1501-YF91	TCM	CAVRVTGGFKTIF-CASSYEAGSSSGANVLTF	1
HD3	1501-YF91	TCM	CIPTL_YALNF-CASSYRPDTEAFF	1
HD3	1501-YF91	TCM	CALSEGDQGGKLIF-CSVEGTSGRGEQFF	1
HD3	1501-YF91	TCM	CAGVGMNRDDKIIF-CASSQDVGGEIGNSPLHF	2
HD3	1501-YF91	TCM	CAGVGMNRDDKIIF-CASSQDVGGEIGNSPLHF	2
HD3	1501-YF91	TCM	CAFVPFGGAQKLVF-CAVFTVEAGRDEAFF	2
HD3	1501-YF91	TCM	CAVGAR_TYKYIF-CASSQDLGFENSPLHF	3
HD3	1501-YF91	TCM	CAVGAR_TYKYIF-CASSQDLGFENSPLHF	3
HD3	1501-YF91	TCM	CIVRVERDDKIIF-CSAWDRANYGYTF	4
HD3	1501-YF91	TCM	CIVRVERDDKIIF-CSAWDRANYGYTF	4
HD3	1501-YF91	TCM	CALSEGDQGGKLIF-CASASVAGGTYEQYF	6
HD3	1501-YF91	TCM	CALSEGDQGGKLIF-CASASVAGGTYEQYF	6
HD3	1501-YF91	TCM	CALSEGDQGGKLIF-CASASVAGGTYEQYF	6
HD3	1501-YF91	TCM	CALSEGDQGGKLIF-CASASVAGGTYEQYF	6
HD3	1501-YF91	TCM	CAAPLSGSARQLTF-CSARGRIANYGYTF	7
HD3	1501-YF91	TCM	CSVEGTSGRGEQFF	12
HD3	1501-YF91	TCM	CSVEGTSGRGEQFF	12
HD3	1501-YF91	TEM	CAASEEMDSSYKLIF-CASKHRPDSYEQYF	1
HD3	1501-YF91	TEM	CIVTFRR_GADGLTF-CASSSQNYGYTF	1
HD3	1501-YF91	TEM	CALSEPGGGSEKLVF-CASSYGMWYGYTF	1
HD3	1501-YF91	TEM	CAAPLSGSARQLTF-CSARGRIANYGYTF	7
HD3	1501-YF91	TEM	CAAPLSGSARQLTF-CSARGRIANYGYTF	7
HD3	1501-YF91	TEMRA	CASSLSAGGRDEQFF	1

Table S5: YF45 tetramer+ T cells before and 14 days after YFV vaccination
* Phenotypes are defined as in Table S3. Green highlights naïve and T_{MN} cells in day 14 samples.

Donor	visit	Phenotype*	CDR	clone size
HD2	d14 post	NA	CIALPAGGTSYGKLTF-CASSPTGLGDYGYTF;CIVRVQTGANNLFF-CASSPTGLGDYGYTF	6
HD2	d14 post	TCM	CILRPNYGGSQGNLIF-CASSKTSGLQSYNEQFF;CILRPNYGGSQGNLIF-CASSKTSGLQSYNEQFF	1
HD2	d14 post	TCM	CIVRPSAGNTGKLIF-CASSLGFSMTDRGYTF;CAENNGQLGDKIIF-CASSLGFSMTDRGYTF	1
HD2	d14 post	TCM	GFGNVLHC-CASSQARQGTPEAFF;CAMREGPFSGGYNKLIF-CASSQARQGTPEAFF	1
HD2	d14 post	TCM	CIVRVAGSARQLTF-CASSSGEIPRSYEQYF	1
HD2	d14 post	TCM	CIVKNTGTASKLTF-CASSVAGTVNTEAFF	1
HD2	d14 post	TCM	CATGPKRGLSDTQYF	1
HD2	d14 post	TCM	CIVRVGNNNDMRF-CSAGGRVGTGELFF;CIVRVGNNNDMRF-CSAGGRVGTGELFF	1
HD2	d14 post	TCM	CSARAITARYEQYF	1
HD2	d14 post	TCM	CAYRSRYPYNTDKLIF-CSARAVVGGYEQYF;CAGGLTQGGSEKLVF-CSARAVVGGYEQYF	1
HD2	d14 post	TCM	CSARLSGSIGEQFF	1
HD2	d14 post	TCM	CSARPGSIETQYF	1
HD2	d14 post	TCM	CIVRVLAGGTSYGKLTF-CSARQGSLNTQYF	1
HD2	d14 post	TCM	CAVSVSYLDGGTSYGKLTF-CSARVLSSYNSPLHF;CAVSVSYLDGGTSYGKLTF-CSARVLSSYNSPLHF	1
HD2	d14 post	TCM	CAVRGRLGGGYNKLIF-CSASPLTGDEETQYF	1
HD2	d14 post	TCM	CAGTNNDMRF-CSASVVTGTFGEQYF;CAGTNNDMRF-CSASVVTGTFGEQYF	1
HD2	d14 post	TCM	CIVRSAGNMLTF-CASSSGASTPGYEQYF;CIVRGAGNMLTF-CASSSGASTPGYEQYF	2
HD2	d14 post	TCM	CIVRVQTGANNLFF-CASTSSGGIYNEQFF	2
HD2	d14 post	TCM	CALIRNSGNTPLVF-CSARPRTGGEQYF;CAMSVTNPFHNAGNMLTF-CSARPRTGGEQYF	2
HD2	d14 post	TCM	CVVSVGKFSDGQKLLF-CASSQRQGINTEAFF	3
HD2	d14 post	TCM	CILKTSGSRLTF-CASSTGSTTPNNEQFF;CILKTSGSRLTF-CASSTGSTTPNNEQFF	3
HD2	d14 post	TCM	CILKTSGSRLTF-CASSTGSTTPNNEQFF;CILKTSGSRLTF-CASSTGSTTPNNEQFF	3
HD2	d14 post	TCM	CIVRSTGNKLTF-CSAIAGPAYNEQFF;CIVRSTGNKLTF-CSAIAGPAYNEQFF	3
HD2	d14 post	TCM	CIVRSTGNKLTF-CSAIAGPAYNEQFF	3
HD2	d14 post	TCM	CIVRVQTGANNLFF-CASSLAVTVRSSNYGYTF;FIVRVQTGANNLFF-CASSLAVTVRSSNYGYTF	4
HD2	d14 post	TCM	CIVRVRAGNTPLVF-CASSLGSLAGSYNEQFF;CIALPAGGTSYGKLTF-CASSLGSLAGSYNEQFF	4
HD2	d14 post	TCM	CIVRVRAGNTPLVF-CASSLGSLAGSYNEQFF	4
HD2	d14 post	TCM	CIVRVRAGNTPLVF-CASSLGSLAGSYNEQFF	4
HD2	d14 post	TCM	CASSSTTDGYTF	4
HD2	d14 post	TCM	CASSSTTDGYTF	4
HD2	d14 post	TCM	CASSSTTDGYTF	4
HD2	d14 post	TCM	CAMTGSARQLTF-CSARAVTGDNSPLHF;CAMTGSARQLTF-CSARAVTGDNSPLHF	4
HD2	d14 post	TCM	CAMTGSARQLTF-CSARAVTGDNSPLHF;CAMTGSARQLTF-CSARAVTGDNSPLHF	4
HD2	d14 post	TCM	CIALPAGGTSYGKLTF-CASSPTGLGDYGYTF;CIALPAGGTSYGKLTF-CASSPTGLGDYGYTF	6
HD2	d14 post	TEM	CIVRSSNTGNQFYF-CASNHRLPYNSPLHF;CIVRSSNTGNQFYF-CASNHRLPYNSPLHF	1
HD2	d14 post	TEM	CIVKGTGTASKLTF-CASSLAGTVNTEAFF;CIVKGTGTASKLTF-CASSLAGTVNTEAFF	1
HD2	d14 post	TEM	CIVRVGYGQNFVF-CASSLQPGQVSYEQYF	1
HD2	d14 post	TEM	CADGSGNTGKLIF-CASSLTQGRNSPLHF	1
HD2	d14 post	TEM	CALSRNSGNTPLVF-CASSQNVGIVYEQYF	1
HD2	d14 post	TEM	CASSSGEIPRSYEQYF	1
HD2	d14 post	TEM	CALVSNSGYALNF-CATSDFPVVGVNYGYTF	1
HD2	d14 post	TEM	CAIPSQFYF-CSANPRVEGTQYF	1
HD2	d14 post	TEM	CVVSAWYSSASKIIF-CSARAVSGGSTDTQYF	1
HD2	d14 post	TEM	CIVRLPISGNTPLVF-CSARPPTLGQTNTEAFF	1
HD2	d14 post	TEM	CALDSRDAGKSTF-CSARRTSILNEQYF	1

HD2	d14 post	TEM	CVVSSYYSSASKIIF-CSARTISGGSTDTQYF;CIVRSTGNKLTF-CSARTISGGSTDTQYF	1
HD2	d14 post	TEM	CIVRVGSWNAGNMLTF-CSASPRVYGEQYF;CIVRVQTGANNLFF-CSASPRVYGEQYF	1
HD2	d14 post	TEM	CIVLTSGTYKYIF-CSASWTSFGEQYF	1
HD2	d14 post	TEM	CSATVRTGGEQYF	1
HD2	d14 post	TEM	CIVSSGNTGKLIF-CSVAWRVGTGELFF	1
HD2	d14 post	TEM	CLVGAGNTGKLIF-CSVSDRVGSDTIYF;GFQKLVF-CSVSDRVGSDTIYF	1
HD2	d14 post	TEM	CIALKAAGNKLTF-CSVSGLAGGGEQFF;CIVRVQTGANNLFF-CSVSGLAGGGEQFF	1
HD2	d14 post	TEM	CALSGNNNARLMF-CSVVVRTGGNQPQHF;CALSGNNNARLMF-CSVVVRTGGNQPQHF	1
HD2	d14 post	TEM	CIVRSAGNMLTF-CASSSGASTPGYEQYF	2
HD2	d14 post	TEM	CIVRVQTGANNLFF-CASTSSGGIYNEQFF	2
HD2	d14 post	TEM	TTRPGYSTLTF-CSAQPHITSEQYF	2
HD2	d14 post	TEM	TTRPGYSTLTF-CSAQPHITSEQYF	2
HD2	d14 post	TEM	CALIRNSGNTPLVF-CSARPRTGGEQYF;CAMSVTNPFHNAGNMLTF-CSARPRTGGEQYF	2
HD2	d14 post	TEM	CVVSVGKFSDGQKLLF-CASSQRQGINTEAFF;CIVRSAGNMLTF-CASSQRQGINTEAFF	3
HD2	d14 post	TEM	CVVSVGKFSDGQKLLF-CASSQRQGINTEAFF	3
HD2	d14 post	TEM	CASSTGGLTTEAFF	3
HD2	d14 post	TEM	CASSTGGLTTEAFF	3
HD2	d14 post	TEM	CASSTGGLTTEAFF	3
HD2	d14 post	TEM	CILKTSGSRLTF-CASSTGSTTPNNEQFF;CILKTSGSRLTF-CASSTGSTTPNNEQFF	3
HD2	d14 post	TEM	CIVRSTGNKLTF-CSAIAGPAYNEQFF	3
HD2	d14 post	TEM	CIVRVQTGANNLFF-CASSLAVTVRSSNYGYTF;CIVRVQTGANNLFF-CASSLAVTVRSSNYGYTF	4
HD2	d14 post	TEM	CIVRVQTGANNLFF-CASSLAVTVRSSNYGYTF	4
HD2	d14 post	TEM	CIVRVQTGANNLFF-CASSLAVTVRSSNYGYTF	4
HD2	d14 post	TEM	CIVRVRAGNTPLVF-CASSLGSLAGSYNEQFF	4
HD2	d14 post	TEM	CASSSTTDGYTF	4
HD2	d14 post	TEM	CAMTGSARQLTF-CSARAVTGDNSPLHF;CAMTGSARQLTF-CSARAVTGDNSPLHF	4
HD2	d14 post	TEM	CAMTGSARQLTF-CSARAVTGDNSPLHF;CAMTGSARQLTF-CSARAVTGDNSPLHF	4
HD2	d14 post	TEM	CIALPAGGTSYGKLTF-CASSPTGLGDYGYTF;CIALPAGGTSYGKLTF-CASSPTGLGDYGYTF	6
HD2	d14 post	TEM	CIALPAGGTSYGKLTF-CASSPTGLGDYGYTF;CIALPAGGTSYGKLTF-CASSPTGLGDYGYTF	6
HD2	d14 post	TEM	CIALPAGGTSYGKLTF-CASSPTGLGDYGYTF	6
HD2	d14 post	TEM	CIALPAGGTSYGKLTF-CASSPTGLGDYGYTF	6
HD3	d14 post	Naive	CAMREVNNARLMF-CASSLGKTTSGNTIYF	1
HD3	d14 post	Naive	CASSPQDSLGVYNEQFF	1
HD3	d14 post	Naive	CIVRNNAGNMLTF-CASSPQGPLINEQFF	26
HD3	d14 post	TCM	CASSFPMNTEAFF	1
HD3	d14 post	TCM	CASSLDYNEQFF	1
HD3	d14 post	TCM	CVVIPNW_ANNLFF-CASSLEAGLSTDTQYF	1
HD3	d14 post	TCM	CAMREGNNFNKFYF-CASSLGGIESGYTF	1
HD3	d14 post	TCM	CIVRPLGGAQKLVF-CASSLGQVLFTDTQYF	1
HD3	d14 post	TCM	CIVREAGGFKTIF-CASSLHGGTVSTEAFF	1
HD3	d14 post	TCM	CIVRVAAISGGYNKLIF-CASSPEGPTYF	1
HD3	d14 post	TCM	CAENRPGV_SSASKIIF-CASSPQDSLGVYNEQFF	1
HD3	d14 post	TCM	CIPPLA_DDKIIF-CASSSRQGLNTEAFF	1
HD3	d14 post	TCM	CASSSVPGPNTEAFF	1
HD3	d14 post	TCM	CAPSTGGGNKLTF-CASSTQGLITEAFF	1
HD3	d14 post	TCM	CAVQAWVTGGGNKLTF-CASSVGIGSTDTQYF	1
HD3	d14 post	TCM	CASTAGPLDTEAFF	1
HD3	d14 post	TCM	CPHP-CSARVLTVGEQYF	1
HD3	d14 post	TCM	CASYRDDKIIF-CSARVLVGNTIYF	1
HD3	d14 post	TCM	CAGPVGSSNTGKLIF-CSVLHRVGGTEAFF	1
HD3	d14 post	TCM	CALFSSNTGKLIF-CSVRRGNGQGDTEAFF	1
HD3	d14 post	TCM	CIVRVYAGNMLTF-CASSLVQLNTEAFF	2
HD3	d14 post	TCM	CIVRSNTDKLIF-CASSPYWTVTDSPLHF;CIWHE_LFYF-CASSPYWTVTDSPLHF	2

HD3	d14 post	TCM	CIVKVQTGANNLFF-CASSSSGGIYNEQFF	2
HD3	d14 post	TCM	CLVGATGSARQLTF-CSARGQGVLGELFF;CLVGATGSARQLTF-CSARGQGVLGELFF	2
HD3	d14 post	TCM	CLVGATGSARQLTF-CSARGQGVLGELFF	2
HD3	d14 post	TCM	CAESVGLH_NAGKSTF-CSASLRTGQNQPQHF	2
HD3	d14 post	TCM	CAESVGLH_NAGKSTF-CSASLRTGQNQPQHF	2
HD3	d14 post	TCM	CALSDGGYQKVTF-CSATTLSGGGRETQYF	2
HD3	d14 post	TCM	CIVRVGAQGAQKLVF-CASSFPRGQNINQPQHF	3
HD3	d14 post	TCM	CIGKNTGTASKLTF-CASSPPGQVNTEAFF	3
HD3	d14 post	TCM	CIVRVGNYGQNFVF-CASSSGLAIEQYF	3
HD3	d14 post	TCM	CALCTGGGNKLTF-CASSSPGLNTEAFF	3
HD3	d14 post	TCM	CALCTGGGNKLTF-CASSSPGLNTEAFF	3
HD3	d14 post	TCM	CALDNAGGTSYGKLTF-CSARVVVGDTQYF	3
HD3	d14 post	TCM	CIVRATGNQFYF-CSARLGNTNNEQFF	4
HD3	d14 post	TCM	CIVRATGNQFYF-CSARLGNTNNEQFF	4
HD3	d14 post	TCM	CASGSSNTGKLIF-CSASLRTGQNQPQHF	4
HD3	d14 post	TCM	CIGGRK_QGNLIF-CASSLAQQGNTEAFF;CIVRVGASNTGKLIF-CASSLAQQGNTEAFF	5
HD3	d14 post	TCM	CIVRVGASNTGKLIF-CASSLAQQGNTEAFF	5
HD3	d14 post	TCM	CIVRVGASNTGKLIF-CASSLAQQGNTEAFF	5
HD3	d14 post	TCM	CSARALSTGEQYF	7
HD3	d14 post	TCM	CSARALSTGEQYF	7
HD3	d14 post	TCM	CSARALSTGEQYF	7
HD3	d14 post	TCM	CSARALSTGEQYF	7
HD3	d14 post	TCM	CSARALSTGEQYF	7
HD3	d14 post	TCM	CSARALSTGEQYF	7
HD3	d14 post	TCM	CIVRNNAGNMLTF-CASSPQGPLINEQFF;CIVRNNAGNMLTF-CASSPQGPLINEQFF	26
HD3	d14 post	TCM	CIVRNNAGNMLTF-CASSPQGPLINEQFF;CIVRNNAGNMLTF-CASSPQGPLINEQFF	26
HD3	d14 post	TCM	CIVRNNAGNMLTF-CASSPQGPLINEQFF	26
HD3	d14 post	TCM	CIVRNNAGNMLTF-CASSPQGPLINEQFF	26
HD3	d14 post	TCM	CIVRNNAGNMLTF-CASSPQGPLINEQFF	26
HD3	d14 post	TCM	CIVRNNAGNMLTF-CASSPQGPLINEQFF	26
HD3	d14 post	TCM	CIVRNNAGNMLTF-CASSPQGPLINEQFF	26
HD3	d14 post	TCM	CIVRNNAGNMLTF-CASSPQGPLINEQFF	26
HD3	d14 post	TCM	CIVRNNAGNMLTF-CASSPQGPLINEQFF	26
HD3	d14 post	TCM	CIVRNNAGNMLTF-CASSPQGPLINEQFF	26
HD3	d14 post	TCM	CIVRNNAGNMLTF-CASSPQGPLINEQFF	26
HD3	d14 post	TCM	CIVRNNAGNMLTF-CASSPQGPLINEQFF	26
HD3	d14 post	TCM	CIVRNNAGNMLTF-CASSPQGPLINEQFF	26
HD3	d14 post	TCM	CAGQGYNQGGKLIF-CSASYLTGSYNEQFF	30
HD3	d14 post	TCM	CAGQGYNQGGKLIF-CSASYLTGSYNEQFF	30
HD3	d14 post	TCM	CAGQGYNQGGKLIF-CSASYLTGSYNEQFF	30
HD3	d14 post	TCM	CAGQGYNQGGKLIF-CSASYLTGSYNEQFF	30
HD3	d14 post	TCM	CAGQGYNQGGKLIF-CSASYLTGSYNEQFF	30
HD3	d14 post	TCM	CAGQGYNQGGKLIF-CSASYLTGSYNEQFF	30
HD3	d14 post	TCM	CAGQGYNQGGKLIF-CSASYLTGSYNEQFF	30
HD3	d14 post	TCM	CAGQGYNQGGKLIF-CSASYLTGSYNEQFF	30
HD3	d14 post	TCM	CAGQGYNQGGKLIF-CSASYLTGSYNEQFF	30
HD3	d14 post	TEM	CASSFGTPLGGELFF	1
HD3	d14 post	TEM	CASSFLKPGQAHYGYTF	1
HD3	d14 post	TEM	CAVRLMNYGGSQGNLIF-CASSLEWRGAQMRPFF	1
HD3	d14 post	TEM	CIVRVSGGGGADGLTF-CASSLGSVPPGNTIYF	1
HD3	d14 post	TEM	CIVKAGTALIF-CASSLNPLAGGLTGELFF	1
HD3	d14 post	TEM	CIVRVAGDTGRRALTF-CASSPNLWVPHEQFF	1
HD3	d14 post	TEM	CASSQGGLNTEAFF	1
HD3	d14 post	TEM	CIVKVQTGANNLFF-CASSQSGGVYNEQFF	1
HD3	d14 post	TEM	CAPGR_DKIIF-CASSRWTGPLGQETQYF	1
HD3	d14 post	TEM	CALLNYGQNFVF-CASSSVPGPNTEAFF	1
HD3	d14 post	TEM	CAMSHTGFQKLVF-CASSVGSVSGGTGELFF;CIVKAGTALIF-CASSVGSVSGGTGELFF	1
HD3	d14 post	TEM	KGRDDKIIF-CASTLSGGLYNEQFF;CIVRPQTGANNLFF-CASTLSGGLYNEQFF	1
HD3	d14 post	TEM	CAAIRGTYKYIF-CSANVRVEGEQYF	1
HD3	d14 post	TEM	CAVEPQLNNAGGTSYGKLTF-CSARARVGGEAFF	1
HD3	d14 post	TEM	CAAPGHSNYQLIW-CSARGRVGGEQYF	1

HD3	d14 post	TEM	CIVRATGNQFYF-CSARQGVVNEQFF;CIVRATGNQFYF-CSARQGVVNEQFF	1
HD3	d14 post	TEM	CAVDNTNAGKSTF-CSARTRVGNSPLHF	1
HD3	d14 post	TEM	CAENRNAGNMLTF-CSASGGSNSGNTIYF	1
HD3	d14 post	TEM	CSATLRTLGEQFF	1
HD3	d14 post	TEM	CATDPYSSNTGKLIF-CSATSLVTSRTGIQQYF	1
HD3	d14 post	TEM	CLVGLPGSSNTGKLIF-CSTLDRVGGGELFF	1
HD3	d14 post	TEM	CIVPGSTPLVF-CSVADRVGTGELFF	1
HD3	d14 post	TEM	CIAFSGNTPLVF-CSVGARVNTGELFF;CIAFSVNTPLVF-CSVGARVNTGELFF	1
HD3	d14 post	TEM	CIVRVSHKAAGNKLTF-CSVGNRWARVVGSGNTIYF	1
HD3	d14 post	TEM	CIVRPLNTGNQFYF-CSVQIGRVGGEQFF;SMVLGANSKLTF-CSVQIGRVGGEQFF	1
HD3	d14 post	TEM	CSVRTGQVTGELFF	1
HD3	d14 post	TEM	CIVRVVTNAGKSTF-CASSLGFMNTEAFF	2
HD3	d14 post	TEM	CIVRVYAGNMLTF-CASSLVQLNTEAFF	2
HD3	d14 post	TEM	CIVRSNTDKLIF-CASSPYWTVTDSPLHF	2
HD3	d14 post	TEM	CIVKVQTGANNLFF-CASSSSGGIYNEQFF	2
HD3	d14 post	TEM	CIVRVQTGANNLFF-CASTKSGGVYNEQFF;CIVRVQTGANNLFF-CASTKSGGVYNEQFF	2
HD3	d14 post	TEM	CIVRVQTGANNLFF-CASTKSGGVYNEQFF	2
HD3	d14 post	TEM	CAQGGRNNYGQNFVF-CSARLRTGWDSPLHF;CLVGATGSARQLTF-CSARLRTGWDSPLHF	2
HD3	d14 post	TEM	CAQGGRNNYGQNFVF-CSARLRTGWDSPLHF	2
HD3	d14 post	TEM	CILGSSNTGKLIF-CSASQRTGSSGNTIYF;CILGSSNTGKLIF-CSASQRTGSSGNTIYF	2
HD3	d14 post	TEM	CALSDGGYQKVTF-CSATTLSGGGRETQYF	2
HD3	d14 post	TEM	CIVRVGAQGAQKLVF-CASSFPRGQNINQPQHF	3
HD3	d14 post	TEM	CIVRVGAQGAQKLVF-CASSFPRGQNINQPQHF	3
HD3	d14 post	TEM	CAGAGGTSYGKLTF-CASSMTVQGAIGANVLTF	3
HD3	d14 post	TEM	CAGAGGTSYGKLTF-CASSMTVQGAIGANVLTF	3
HD3	d14 post	TEM	CAGAGGTSYGKLTF-CASSMTVQGAIGANVLTF	3
HD3	d14 post	TEM	CIGKNTGTASKLTF-CASSPPGQVNTEAFF	3
HD3	d14 post	TEM	CIGKNTGTASKLTF-CASSPPGQVNTEAFF	3
HD3	d14 post	TEM	CIVRVGNYGQNFVF-CASSSGLAIEQYF	3
HD3	d14 post	TEM	CIVRVGNYGQNFVF-CASSSGLAIEQYF	3
HD3	d14 post	TEM	CALCTGGGNKLTF-CASSSPGLNTEAFF	3
HD3	d14 post	TEM	CASSTQGLITEAFF	3
HD3	d14 post	TEM	CIVRATGNQFYF-CSARLGNTNNEQFF	4
HD3	d14 post	TEM	CIVRATGNQFYF-CSARLGNTNNEQFF	4
HD3	d14 post	TEM	CASGSSNTGKLIF-CSASLRTGQNQPQHF	4
HD3	d14 post	TEM	CASGSSNTGKLIF-CSASLRTGQNQPQHF	4
HD3	d14 post	TEM	CASGSSNTGKLIF-CSASLRTGQNQPQHF	4
HD3	d14 post	TEM	CIVRVGASNTGKLIF-CASSLAQQGNTEAFF	5
HD3	d14 post	TEM	CIVRVGASNTGKLIF-CASSLAQQGNTEAFF	5
HD3	d14 post	TEM	CSARALSTGEQYF	7
HD3	d14 post	TEM	CIVRNNAGNMLTF-CASSPQGPLINEQFF	26
HD3	d14 post	TEM	CIVRNNAGNMLTF-CASSPQGPLINEQFF	26
HD3	d14 post	TEM	CIVRNNAGNMLTF-CASSPQGPLINEQFF	26
HD3	d14 post	TEM	CIVRNNAGNMLTF-CASSPQGPLINEQFF	26
HD3	d14 post	TEM	CIVRNNAGNMLTF-CASSPQGPLINEQFF	26
HD3	d14 post	TEM	CIVRNNAGNMLTF-CASSPQGPLINEQFF	26
HD3	d14 post	TEM	CIVRNNAGNMLTF-CASSPQGPLINEQFF	26
HD3	d14 post	TEM	CIVRNNAGNMLTF-CASSPQGPLINEQFF	26
HD3	d14 post	TEM	CIVRNNAGNMLTF-CASSPQGPLINEQFF	26
HD3	d14 post	TEM	CIVRNNAGNMLTF-CASSPQGPLINEQFF	26
HD3	d14 post	TEM	CIVRNNAGNMLTF-CASSPQGPLINEQFF	26
HD3	d14 post	TEM	CAGQGYNQGGKLIF-CSASYLTGSYNEQFF	30
HD3	d14 post	TEM	CAGQGYNQGGKLIF-CSASYLTGSYNEQFF	30
HD3	d14 post	TEM	CAGQGYNQGGKLIF-CSASYLTGSYNEQFF	30
HD3	d14 post	TEM	CAGQGYNQGGKLIF-CSASYLTGSYNEQFF	30
HD3	d14 post	TEM	CAGQGYNQGGKLIF-CSASYLTGSYNEQFF	30
HD3	d14 post	TEM	CAGQGYNQGGKLIF-CSASYLTGSYNEQFF	30
HD3	d14 post	TEM	CAGQGYNQGGKLIF-CSASYLTGSYNEQFF	30
HD3	d14 post	TEM	CAGQGYNQGGKLIF-CSASYLTGSYNEQFF	30
HD3	d14 post	TEM	CAGQGYNQGGKLIF-CSASYLTGSYNEQFF	30
HD3	d14 post	TEM	CAGQGYNQGGKLIF-CSASYLTGSYNEQFF	30
HD3	d14 post	TEM	CAGQGYNQGGKLIF-CSASYLTGSYNEQFF	30

HD3	d14 post	TEM	CAGQGYNQGGKLIF-CSASYLTGSYNEQFF	30
HD3	d14 post	TEM	CAGQGYNQGGKLIF-CSASYLTGSYNEQFF	30
HD3	d14 post	TEM	CAGQGYNQGGKLIF-CSASYLTGSYNEQFF	30
HD3	d14 post	TEM	CAGQGYNQGGKLIF-CSASYLTGSYNEQFF	30
HD3	d14 post	TEM	CAGQGYNQGGKLIF-CSASYLTGSYNEQFF	30
HD3	d14 post	TEMRA	CALTPLNSGGSNYKLTF-CSAVVGSVMRSPLHF	1
HD3	d14 post	TEMRA	CIVRVVTNAGKSTF-CASSLGFMNTEAFF	2
HD3	d14 post	TEMRA	CASSTQGLITEAFF	3
HD3	d14 post	TEMRA	CIVRNNAGNMLTF-CASSPQGPLINEQFF	26
HD3	d14 post	TEMRA	CAGQGYNQGGKLIF-CSASYLTGSYNEQFF	30
HD3	d14 post	TEMRA	CAGQGYNQGGKLIF-CSASYLTGSYNEQFF	30
HD3	d14 post	TEMRA	CAGQGYNQGGKLIF-CSASYLTGSYNEQFF	30
HD3	d14 post	TMN	CSARVLAGGPGEQYF	1
HD3	d14 post	TMN	CASSTQGLITEAFF	3
HD3	d14 post	TSCM	CIVRVAVNAGNMLTF-CSVGQASNTGELFF	1
HD3	d14 post	TSCM	CAGQGYNQGGKLIF-CSASYLTGSYNEQFF	30
HD3	d14 post	TSCM	CAGQGYNQGGKLIF-CSASYLTGSYNEQFF	30
HD2	pre-vac	NA	CILGRDDKIIF-CSAPILAGQGQKLFF;CILGRDDKIIF-CSAPILAGQGQKLFF	1
HD2	pre-vac	NA	CAVAQGGSEKLVF-CSARVLVGYEQYF	1
HD2	pre-vac	Naive	CAASVAGTYKYIF-CASRDRFRTDTQYF	1
HD2	pre-vac	Naive	CASREGPGPRNKNYGYTF	1
HD2	pre-vac	Naive	CIVISGAGGTSYGKLTF-CASRKGTGTNEKLFF	1
HD2	pre-vac	Naive	CIVRVAGSGGYQKVTF-CASSEQPLDYGYTF	1
HD2	pre-vac	Naive	CAMRGNQAGTALIF-CASSLASAQETQYF	1
HD2	pre-vac	Naive	CAVGRSGGSYIPTF-CASSPVVLANQETQYF;CAVGRSGGSYIPTF-CASSPVVLANQETQYF	1
HD2	pre-vac	Naive	CIVRVAGIGGNTPLVF-CASSQVPGGSEQYF;CIVRVAGIGGNTPLVF-CASSQVPGGSEQYF	1
HD2	pre-vac	Naive	CASSTFGQGSTGNTEAFF	1
HD2	pre-vac	Naive	CAMSPNYGNNRLAF-CASSYKENYGYTF	1
HD2	pre-vac	Naive	CIVRVAVLSGANNLFF-CASWGNYGYTF;CIVRVSVLSGANNLFF-CASWGNYGYTF	1
HD2	pre-vac	Naive	CVVLKPDNFNKFYF-CATSEGPFRRNIQYF	1
HD2	pre-vac	Naive	CATVGTGGDVRGDEAFF	1
HD2	pre-vac	Naive	CVVSDFKDKLSF-CSAIVLVSGEQYF;CVVSDFKDKLSF-CSAIVLVSGEQYF	1
HD2	pre-vac	Naive	CAFGDSNYQLIW-CSAKMLTGNQPQHF	1
HD2	pre-vac	Naive	CVVSGGYSGNTGKLIF-CSAKVLAAFQETQYF;CVVSGGYSGNTGKLIF-CSAKVLAAFQETQYF	1
HD2	pre-vac	Naive	CSAPVIAGRADTQYF	1
HD2	pre-vac	Naive	CAVYDYKLSF-CSARAGTGAFEQYF	1
HD2	pre-vac	Naive	CAGLNTGGFKTIF-CSARASRIGTDTQYF;CAGLNTGGFKTIF-CSARASRIGTDTQYF	1
HD2	pre-vac	Naive	CIVRVEANAGGTSYGKLTF-CSARDLTILTDTQYF	1
HD2	pre-vac	Naive	CALSAAGGTSYGKLTF-CSARGVVGRNSPLHF	1
HD2	pre-vac	Naive	CAVRSYNTDKLIF-CSARILAGGPYNEQFF	1
HD2	pre-vac	Naive	CAYRSGYRDDKIIF-CSARLRVSSETQYF	1
HD2	pre-vac	Naive	CVVSDSGNQFYF-CSARPLTNSPLHF;CVVSDSGNQFYF-CSARPLTNSPLHF	1
HD2	pre-vac	Naive	CSARPLTTMKTQYF	1
HD2	pre-vac	Naive	CIVRPPTGNQFYF-CSARRAVISYEQYF;CAFRKASGTYKYIF-CSARRAVISYEQYF	1
HD2	pre-vac	Naive	CAPRDSGGYQKVTF-CSARVGANYGYTF	1
HD2	pre-vac	Naive	CAVRPRSGNTPLVF-CSARVGSIMDTQYF	1
HD2	pre-vac	Naive	CAYRTGNTGKLIF-CSARVGSISTLHF	1
HD2	pre-vac	Naive	CAVRSPGGGADGLTF-CSARVGSLLAQYF	1
HD2	pre-vac	Naive	CAVNQAGTALIF-CSARVGSLNTIYF;CAVNQAGTALIF-CSARVGSLNTIYF	1
HD2	pre-vac	Naive	CAVPSGSARQLTF-CSARVGSLQETQYF	1
HD2	pre-vac	Naive	CSARVGSLQETQYF	1
HD2	pre-vac	Naive	CAGPGGGADGLTF-CSARVGSLTAQYF	1
HD2	pre-vac	Naive	CAVRGLSGGSNYKLTF-CSARVGSLTDTQYF;CAVRGLSGGSNYKLTF-CSARVGSLTDTQYF	1
HD2	pre-vac	Naive	CVVRANSNSGYALNF-CSARVGSLVETQYF;CVVRANSNSGYALNF-CSARVGSLVETQYF	1
HD2	pre-vac	Naive	CAVKGYNTDKLIF-CSARVLAGGPGETQYF	1
HD2	pre-vac	Naive	CSARVLAGHPGETQYF	1
HD2	pre-vac	Naive	CAAFFSDGQKLLF-CSARVLAGRADTQYF;CAAFFSDGQKLLF-CSARVLAGRADTQYF	1

HD2	pre-vac	Naive	CAVSERTGGTSYGKLTF-CSARVLAGRPQETQYF;CAVSERTGGTSYGKLTF-CSARVLAGRPQETQYF	1
HD2	pre-vac	Naive	CAVKDNYGQNFVF-CSARVLAGVGEQFF	1
HD2	pre-vac	Naive	CAVRASTDSWGKLQF-CSARVLAGVSTDTQYF;CAVFWW_YNKLIF-CSARVLAGVSTDTQYF	1
HD2	pre-vac	Naive	CAAINTNAGKSTF-CSARVLIGQETQYF;CAINTLAGGTSYGKLTF-CSARVLIGQETQYF	1
HD2	pre-vac	Naive	CSARVLSGGVRETQYF	1
HD2	pre-vac	Naive	CAAMDSNYQLIW-CSARVLTGNQPQHF	1
HD2	pre-vac	Naive	CAVSVAGGADGLTF-CSARVVINSPLHF;CAVSVAGGADGLTF-CSARVVINSPLHF	1
HD2	pre-vac	Naive	CATDVNAGGTSYGKLTF-CSARVVVEGPQHF	1
HD2	pre-vac	Naive	CAASPLNAGGTSYGKLTF-CSARVVVNQPQHF	1
HD2	pre-vac	Naive	CGAGGTSYGKLTF-CSASLRVYGEQFF;CGAGGTSYGKLTF-CSASLRVYGEQFF	1
HD2	pre-vac	Naive	CASNTGFQKLVF-CSASPLVGNTQYF	1
HD2	pre-vac	Naive	CAVGLDARLMF-CSASVLQGAFEQYF	1
HD2	pre-vac	Naive	CAVETPYNTDKLIF-CSAWVLVQGAQYF	1
HD2	pre-vac	Naive	CSGLVVSARPDTQYF	1
HD2	pre-vac	Naive	CAASKRGSGNTGKLIF-CSVGMGTVTTYEQYF	1
HD2	pre-vac	Naive	CIVRVATYGQNFVF-CSVPSGGDGYTF	1
HD2	pre-vac	Naive	CSVTDRVGSYEQYF	1
HD2	pre-vac	Naive	CAVGGYYGGATNKLIF-CSVVFRVEGGYTF	1
HD2	pre-vac	TCM	CASSASGTSLYEQYF	1
HD2	pre-vac	TCM	CASSLDVVTDTQYF	1
HD2	pre-vac	TCM	CAASASYNTDKLIF-CASSLGVDEQYF	1
HD2	pre-vac	TCM	CIVRGLAAQKLVF-CASSPFLGPGVQPQHF;CIVRGLAAQKLVF-CASSPFLGPGVQPQHF	1
HD2	pre-vac	TCM	CAFMKRGNNARLMF-CASSPGGYRQETQYF	1
HD2	pre-vac	TCM	CAVISNAGGTSYGKLTF-CASSQDKLRGGETQYF	1
HD2	pre-vac	TCM	CAASLMDSNYQLIW-CASSRAVQPGQGATNEKLFF	1
HD2	pre-vac	TCM	CALAVTTGNQFYF-CASSRTGWYEQYF	1
HD2	pre-vac	TCM	CIVRGLNAGNMLTF-CSAALLGTGGGGYTF;CIVRGLAAQKLVF-CSAALLGTGGGGYTF	1
HD2	pre-vac	TCM	CAGSVYNTDKLIF-CSARSRVGGAQHF	1
HD2	pre-vac	TCM	CALKAAGNKLTF-CSARTGSIYNSPLHF	1
HD2	pre-vac	TCM	CAGRPNAGGTSYGKLTF-CSARVGSLPDTQYF	1
HD2	pre-vac	TCM	CVVTNSNSGYALNF-CSARVGSLTDTQYF;CVVTNSNSGYALNF-CSARVGSLTDTQYF	1
HD2	pre-vac	TCM	CALPQGAQKLVF-CSARVGTNYGYTF;CALPQGAQKLVF-CSARVGTNYGYTF	1
HD2	pre-vac	TCM	CAASSYYNDYKLSF-CSASGVAGGEDGYTF	1
HD2	pre-vac	TEM	CFTFSGGYQKVTF-CASSFTTDTQYF	1
HD2	pre-vac	TEM	CAMTSTGFQKLVF-CASSGRLAPNEQFF;CAMTSTGFQKLVF-CASSGRLAPNEQFF	1
HD2	pre-vac	TEM	CILRAVLLGNNNDMRF-CASSMWARWPNTEAFF;CILRAVLLGNNNDMRF-CASSMWARWPNTEAFF	1
HD2	pre-vac	TEM	CASSPGLAGSGTGELFF	1
HD2	pre-vac	TEM	CAYRSAGAQKLVF-CASSPVLHTQYF;CAYRSAGAQKLVF-CASSPVLHTQYF	1
HD2	pre-vac	TEM	CAGPNSNSGYALNF-CSARAIAFGGEQYF	1
HD2	pre-vac	TEM	CALGGSQGNLIF-CSARDLGLAEETQYF;CAVYDYKLSF-CSARDLGLAEETQYF	1
HD2	pre-vac	TEM	CAVEDPLTTDSWGKLQF-CSARVLPSTDTQYF	1
HD2	pre-vac	TEM	CSARVVTGIGQPQHF	1
HD2	pre-vac	TEMRA	CASSLERAPWEKLFF	1
HD2	pre-vac	TEMRA	CAVRVPSWGKLQF-CSAAPGDSYNEQFF	1
HD3	pre-vac	NA	CASGFLAPGQTSGNTIYF	1
HD3	pre-vac	NA	CILSQGGSQGNLIF-CASRPRQTEQFF;CILSQGGSQGNLIF-CASRPRQTEQFF	1
HD3	pre-vac	NA	CIVRVGYNARLMF-CASSQVTQLSTEAFF	1
HD3	pre-vac	NA	CIVRVYTGNQFYF-CASSRDLIGTEAFF	1
HD3	pre-vac	NA	CAPQMDSSYKLIF-CASSVQGLNTEAFF	1
HD3	pre-vac	NA	CAERRGSGGSNYKLTF-CASTPSRTGGYNEQFF	1
HD3	pre-vac	NA	CAFLMDSNYQLIW-CATRLAGGLQGYNEQFF	1
HD3	pre-vac	NA	CSAGGRPGEQYF	1
HD3	pre-vac	NA	CIVMSGNTPLVF-CSAGQRTNTGELFF	1
HD3	pre-vac	NA	CAVRPYDSWGKLQF-CSAPPLSGGQETQYF;CAVGPYDSWGKLQF-CSAPPLSGGQETQYF	1

HD3	pre-vac	NA	CVVSSPNSGYALNF-CSAPTRVEGPQHF;CVVSSPNSGYALNF-CSAPTRVEGPQHF	1
HD3	pre-vac	NA	CAASAGGGAGGTSYGKLTF-CSARALTVYNSPLHF;CAASAGGGAGGTSYGRLTF-CSARALTVYNSPLHF	1
HD3	pre-vac	NA	CAVRGAGGTSYGKLTF-CSARVGSIQETQYF	1
HD3	pre-vac	NA	CAASGAGGTSYGKLTF-CSARVIAGAYEQYF	1
HD3	pre-vac	NA	CALSAPRAQKLVF-CSARVLAGGPRDTQYF	1
HD3	pre-vac	NA	CAVIDYNQGGKLIF-CSARVRADYNSPLHF;CAHGRGSQGNLIF-CSARVRADYNSPLHF	1
HD3	pre-vac	NA	CIALNAGGTSYGKLTF-CSARVRTDLGSPLHF	1
HD3	pre-vac	NA	CGADYDNDMRF-CSARVRVRGTQYF;CGADYDNDMRF-CSARVRVRGTQYF	1
HD3	pre-vac	NA	CAMAGNRDDKIIF-CSARVVAWNSPLHF;LGPDSWGKLQF-CSARVVAWNSPLHF	1
HD3	pre-vac	NA	CAVGTGGTSYGKLTF-CSARVVTGDSPLHF	1
HD3	pre-vac	NA	CAVRAANQAGTALIF-CSPVGTGDEKLFF	1
HD3	pre-vac	NA	CIVRGGKLIF-CSVEMAVRSGEQYF	1
HD3	pre-vac	NA	CILGSSNTGKLIF-CSASQRTGSSGNTIYF;CILGSSNTGKLIF-CSASQRTGSSGNTIYF	2
HD3	pre-vac	NA	CSASVVTGDQPQHF	2
HD3	pre-vac	NA	CSASVVTGDQPQHF	2
HD3	pre-vac	NA	CAVLYSGAGSYQLTF-CSASVVTGDQPQHF	4
HD3	pre-vac	NA	CAVLYSGAGSYQLTF-CSASVVTGDQPQHF	4
HD3	pre-vac	Naive	CAVRDAYGGSQGNLIF-CASSLGASGGAAGEQFF	1
HD3	pre-vac	Naive	CAAGGRGGNTGKLIF-CSALVRTGDQQPQHF	1
HD3	pre-vac	Naive	CAYRAGNQFYF-CSARVGSLLETQYF	1
HD3	pre-vac	Naive	CAVQGTGGFKTIF-CSARVGSLQETQYF	1
HD3	pre-vac	Naive	CSARVGSVQPHEQYF	1
HD3	pre-vac	Naive	CAVLNRDDKIIF-CSARVGSVVGNTIYF	1
HD3	pre-vac	Naive	CAVEEAAGNKLTF-CSARVLAGASGETQYF	1
HD3	pre-vac	Naive	CAGSPQGGSEKLVF-CSARVLTGGNSPLHF	1
HD3	pre-vac	Naive	CAGPYAGGTSYGKLTF-CSARVVTGDNSPLHF	1
HD3	pre-vac	Naive	CALILLLDSNYQLIW-CSARVVTGSDQPQHF	1
HD3	pre-vac	Naive	CIVRVQGGVGNQFYF-CSATTRVEGSQYF	1
HD3	pre-vac	Naive	CSVEGTSGRGEQFF	1
HD3	pre-vac	Naive	CAVSSSNTGKLIF-CSVQHRVGGEQFF	1
HD3	pre-vac	Naive	RSVEGTSGRGEQFF	1
HD3	pre-vac	Naive	CALDNAGGTSYGKLTF-CSARVVVGDTQYF	3
HD3	pre-vac	Naive	CALDNAGGTSYGKLTF-CSARVVVGDTQYF	3
HD3	pre-vac	Naive	CAVLYSGAGSYQLTF-CSASVVTGDQPQHF;CIVRA*YGNKLVF-CSASVVTGDQPQHF	4
HD3	pre-vac	Naive	CAVLYSGAGSYQLTF-CSASVVTGDQPQHF	4

Table S6: Longitudinal follow-up visits

ID	Sex	Total YFV doses	Vaccine dates	Longitudinal visit dates				
HD1	M	2	8/25/2016, 9/8/2017	8/28/17	12/4/18			
HD2	F	2	9/12/2016, 5/26/2017	5/12/17	4/11/19	12/14/21	5/25/23	
HD3	M	2	1/4/2017, 8/11/2017	8/2/17	8/29/19	6/15/23		
HD4	M	1	5/22/17	8/14/17	4/23/18	1/30/20	9/8/21	
HD5	F	1	4/21/17	6/22/17	2/7/19	2/13/20	9/27/21	4/6/23

Table S7: List of flow cytometry antibodies

Number	Specificity	Vendor	Cat#	Fluorochrome	Clone ID	RRID
1	CD4	Biolegend	344634	Brilliant Violet 510	SK3	AB_2566017
2	CD11b	Biolegend	301342	APC/Cyanine7	ICRF44	AB_2563395
3	CD19	Biolegend	302218	APC/Cyanine7	HIB19	AB_314247
4	CD3	Biolegend	300410	PE/Cyanine5	UCHT1	AB_314064
5	CD45RO	Biolegend	304238	Brilliant Violet 605	UCHL1	AB_2562153

6	CCR7	Biolegend	353234	Brilliant Violet 650	G043H7	AB_2563867
7	CXCR3	Biolegend	353716	Brilliant Violet 421	G025H7	AB_11124720
8	CD11a	Biolegend	301216	Alexa Fluor® 488	HI111	AB_2265103
9	CD49d	Biolegend	304332	Brilliant Violet 711	9F10	AB_2687197
10	CD95	Biolegend	305646	Brilliant Violet 785	DX2	AB_2629741
11	CXCR3	Biolegend	353736	PE/Dazzle™ 594	G025H7	AB_2564287
12	CD49d	Biolegend	304322	Brilliant Violet 421	9F10	AB_2563971
13	CD4	Biolegend	300514	APC	RPA-T4	AB_314082
14	TNF-α	Biolegend	502930	PE/Cyanine7	MAb11	AB_2204079
15	IFN-γ	Biolegend	502506	FITC	4S.B3	AB_315230
16	IFN-γ	Biolegend	502538	Brilliant Violet 650	4S.B3	AB_11126140
17	IL-2	Biolegend	500338	Brilliant Violet 510	MQ1-17H12	AB_2562629
